# Screening Oil Components for Interleukin-2-Loaded Lipid-Based Formulations with Molecular Dynamics, In Vitro Characterization, and Cell Culture Evaluation

**DOI:** 10.1021/acsomega.5c12065

**Published:** 2026-02-26

**Authors:** Seval Olgac, Abdurrahman Olgac, Gamze Varan, Zeynep Safak Teksin

**Affiliations:** † Department of Pharmaceutical Technology, Faculty of Pharmacy, 111342 Gazi University, Ankara 06560, Türkiye; ‡ Department of Pharmaceutical Chemistry, Faculty of Pharmacy, Gazi University, Ankara 06560, Türkiye; § Laboratory of Molecular Modeling, Evias Pharmaceutical R&D Ltd, Gazi Teknopark, Ankara 06830, Türkiye; ∥ Department of Vaccine Technology, Vaccine Institute, 37515 Hacettepe University, Ankara 06100, Türkiye

## Abstract

Interleukin-2 (IL-2) is an immunostimulatory cytokine that stimulates T cells, natural killer cells, and other leukocytes, functioning as a growth factor. IL-2 interacts with IL-2Rα, IL-2Rβ, and γ_c_ receptors. IL-2 mediates its therapeutic effects by interacting with the β and γ receptor subunits against cancer, whereas interaction with the α, β, and γ receptor complexes is critical for treating autoimmune disorders. Current efforts aim to develop improved IL-2 biobetters that reduce toxicity through lower dosing strategies, particularly by blocking or slowing the interaction with IL-2Rα. According to these strategies, this study aimed to design a lipid-based IL-2 formulation that could modulate or partially prevent IL-2Rα binding, thereby enhancing the βγ-mediated antitumor efficacy while minimizing α-associated immune activation. Molecular dynamics (MD) simulates the physical motions of atoms and molecules in large systems containing thousands of atoms and is widely used in biotechnological drug formulations. In this study, MD was used to simulate time-dependent interactions between IL-2 and excipients of the lipid-based formulations to determine suitable excipients. Desmond was used to simulate and observe the temporal interactions between the formulation contents and IL-2. Interactions with Arg38, Phe42, and Leu72—key residues of the α-subunit interface—were specifically examined. According to the simulation analyses, polar side chains were protected by lipids, while no incompatibility was expected for the selected excipients. Interactions were observed with Arg38, which interacts with IL-2Rα; thus, an enhanced antitumor effect might be achieved. In addition to the in silico studies, in vitro cell culture experiments were conducted to examine the biological activity and anticancer efficacy of IL-2-loaded nanoemulsions. These studies demonstrated that IL-2’s biological activity was preserved, and its anticancer effect was enhanced against renal carcinoma cells. Overall, the results suggest that the formulation stabilizes IL-2 and enhances its α-targeted antitumor mechanism through rational excipient–protein interactions.

## Graphical abstract

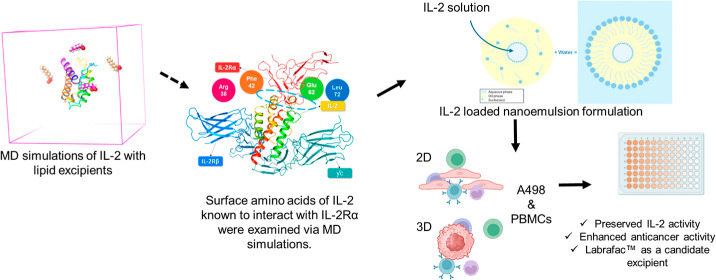

## 1 Introduction

Therapeutic protein drugs play an increasingly important role in current therapeutic strategies. Advances in protein engineering have substantially expanded their therapeutic applications.^1^ Such peptide- and protein-based therapeutics are produced by using biotechnological methods such as recombinant DNA technology. These therapeutics are widely used in the treatment of various cancers, metabolic disorders, autoimmune conditions, and neurodegenerative diseases.^2^

Interleukin-2 (IL-2) was the initial cytokine authorized by the FDA for oncological indications.^3^ Research into the IL-2 cytokine family has highlighted their immunomodulatory roles and antitumor efficacy. Particularly, IL-2 is significant in stimulating antineoplastic immunity within the cancerous tissue milieu.^4^ T cells are the primary source of IL-2 secretion (CD4+ and CD8+) following antigen stimulation, but it can also be released in smaller amounts from other immune cell types, comprising dendritic cells, mast cells, and natural killer T cells.^3^

IL-2 interacts with the IL-2Rα, IL-2Rβ, and γ_c_ subunits. In cancer therapy, its activity is mainly mediated through binding to β and γ subunits, whereas engagement of the full αβγ receptor complex is more relevant in autoimmune disorders (Figure 1).^3,5,6^ Current research aims to design improved IL-2 therapies that maintain efficacy while reducing toxicity, often through a lower dosing regimens. Strategies include the development of novel small chemical entities or antibodies that prevent binding to IL-2Rα, as well as the use of alternative formulation components to modulate this binding.

**Figure 1:**
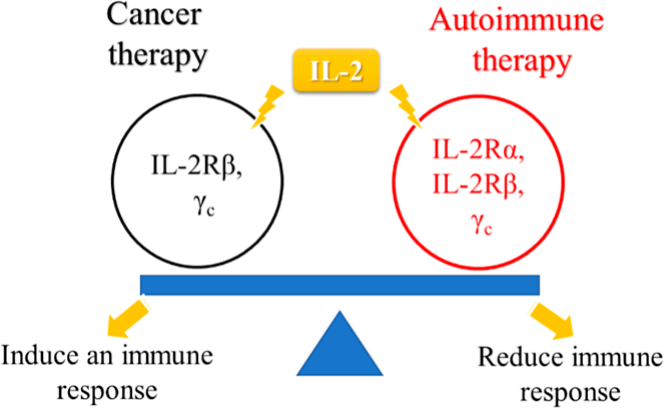
IL-2 binds to different receptor types and balances its roles; β and γ_c_ receptors for anticancer therapy or α, β, and γ_c_ receptors for autoimmune diseases.^5^

Aldesleukin is a therapeutic version of IL-2 produced by a recombinant DNA technology. The drug was engineered with minor modifications to the IL-2 sequence, including (i) removal of the N-terminal alanine and (ii) C125S substitution. It was administered through intravenous infusion or subcutaneously for years. Its clinical use includes treating metastatic renal cell carcinoma and metastatic melanoma.

A major problem is its low oral bioavailability, and due to its protein structure, the parenteral route is preferred. When administered intravenously, recombinant IL-2 exhibits biphasic pharmacokinetics consistent with a two-compartment model. After intravenous bolus or short infusion, IL-2 levels initially decline rapidly with a distribution half-life on the order of ∼10–15 min, followed by a slower elimination phase with a half-life of roughly 60–90 min^7^ When administered subcutaneously, IL-2 becomes detectable in the serum shortly after injection and has an apparent half-life of approximately 1 h.^8^ Pharmaceutical companies have been trying to produce more effective and safer IL-2 using different strategies. In one of the studies conducted for this purpose, mutant IL-2 was produced, and it was shown that these molecules saved their IL-Rβγ binding properties while not binding to IL-2Rα. Therefore, they have been shown to maintain their ability to induce and boost natural killer cells and CD8^+^ effector T cells via IL-2Rβγ signaling in both peripheral tissues and the tumor microenvironment.^9^ In another study, pegylated IL-2 was produced, which has a longer residence time in the body due to pegylation. This molecule was designed as a prodrug and contains six releasable poly­(ethylene glycol) (PEG) chains covalently attached to IL-2 via hydrolyzable ester linkages. Under physiological conditions, these ester bonds undergo slow, nonenzymatic hydrolysis, resulting in the gradual and stepwise release of PEG chains in vivo. This controlled de-PEGylation process leads to the progressive activation of IL-2 over time. As a result, the molecule increases tumor exposure to conjugated IL-2 compared to aldesleukin, while simultaneously extending systemic circulation and modulating receptor engagement.^10^ Also, there have been studies that produced a different compound that mimics IL-2. Protein mimics offer promise for the development of protein-based therapeutics and may reduce side effects by providing improved therapeutic properties. By improving such properties, it is possible to develop biosuperior molecules.^11^

Applying noninvasive methods, especially the oral route, is challenging when administering such drugs to provide effective treatment. Therapeutic peptides/proteins are rapidly metabolized by proteases in the gastrointestinal tract due to their biological structure.^12^ To address this situation, various formulation approaches are applied, such as designing oral encapsulated nanoformulations and adding enzyme inhibitors and absorption enhancers. In our study, building upon these strategies, an orally administered IL-2 formulation was developed.

In recent years, nanoemulsions have emerged as promising formulation strategies for peptide- and protein-based therapeutics. Their nanoscale droplet size provides favorable stability characteristics and makes them suitable carriers for sensitive biomolecules.

Considering the high cost of formulation studies involving recombinant proteins, an in silico prescreening approach is particularly advantageous. Therefore, physics-based molecular dynamics (MD) simulations were employed to virtually screen suitable formulation excipients for nanoemulsion-based IL-2 formulation studies.^13^

Such in silico approaches may help enhance functional bioactivity at a given active pharmaceutical ingredient concentration, which is particularly relevant in the context of IL-2’s narrow therapeutic index. In this study, the approach focused on the rational selection of formulation excipients by investigating their effects on IL-2 interaction with its receptor, particularly IL-2Rα, and associated anticancer activity while also assessing potential permanent binding or incompatibility with IL-2.

In cancer research, cell culture studies are often preferred. Three-dimensional (3D) tumor-immune coculture models provide a more physiologically relevant in vitro platform for evaluating immune-mediated anticancer effects compared with conventional two-dimensional (2D) cultures. 3D cell cultures successfully replicate extracellular matrix organization allowing immune cells to interact with and attack tumor cells in a manner more representative of in vivo conditions.^14^ Accordingly, 3D culture systems are widely used in cancer and stem cell research to investigate complex cellular interactions within the tumor immune microenvironment and to evaluate potential therapeutic strategies.^15^

Despite extensive efforts to engineer safer IL-2 variants or conjugates, comparatively limited attention has been given to formulation-based strategies that aim to modulate IL-2 receptor engagement without alteration of the protein sequence. In particular, the rational selection of formulation excipients capable of transiently interacting with IL-2 surface residues relevant to IL-2Rα binding remains underexplored.

In this study, a multiphase water-in-oil-in-water SNEDDS-derived nanoemulsion system was specifically designed to accommodate and stabilize a hydrophilic protein payload, such as IL-2. While the self-emulsifying behavior facilitates dispersion upon dilution, the presence of an internal aqueous phase enables the effective incorporation of the cytokine.

The primary contribution of this work is to demonstrate a rational, molecular-level framework for the biologic formulation design. In this approach, molecular dynamics (MD) simulations were employed as an excipient screening and decision-guiding tool rather than as a post hoc supportive analysis. This strategy is particularly advantageous, given the high cost of recombinant IL-2 and the need for specialized production technologies.

Accordingly, this study aims to identify compatible excipients through molecular dynamics-guided screening and to develop an IL-2-loaded water-in-oil-in-water SNEDDS-derived nanoemulsion capable of modulating the IL-2Rα interaction. The study further evaluates whether this rationally designed formulation preserves IL-2 biological activity and enhances anticancer efficacy using in vitro cell culture models.

## 2 Materials and Methods

This study was planned to select the most appropriate nanoemulsion excipients that would not show incompatibility and increase the anticancer effect using MD simulations in the development of oral nanodrug delivery systems containing recombinant human IL-2 (rhIL-2). We carried out studies toward the development of an innovative biotechnological drug formulation.

### 2.1 Materials

Labrafac Lipophile WL 1349 was a gift from Gattefossé. Polyglycerol polyricinoleate was purchased from Smart Kimya. Kolliphor EL was purchased from Sigma-Aldrich. rhIL-2 was provided from Elabscience. All cell culture reagents, including fetal bovine serum (FBS), penicillin/streptomycin, RPMI-1640 medium, and Eagle’s Modified Eagle’s Medium (EMEM), were obtained from Gibco. WST-1 Reagent (Cell Proliferation Assay) was obtained from Roche. Phorbol 12-myristate 13-acetate (PMA) was obtained from Cayman Chemical. Matrigel Matrix for 3D cell culture and Transwell insert (5 μm) were purchased from Corning. Lactate Dehydrogenase (LDH) Assay Kit was obtained from BioVision. A498 Human Renal Carcinoma Cells and Human Peripheral Blood Mononuclear Cells (PBMCs) were provided by established cell culture laboratories and used in this study.

### 2.2 Crystal Analysis

Different crystal structures of hIL-2 were systematically evaluated from the Protein Data Bank (PDB) to determine an appropriate starting structure for molecular dynamics simulations (Table 1). As summarized in Table 1, various forms and complexes of IL-2 are publicly available. Among the available native IL-2 structures, PDB entries 1M47 and 1M4C^16^ were considered. Based on its higher resolution and representative native conformation, the 1M47 structure was selected for subsequent simulations. The IL-2 structure’s sequence and secondary structures are represented in Figures 2, and 3D form together with IL-2R is represented in Figure 3. Interacting and structurally relevant residues of IL-2 and IL-2R were also specified and reported in another study (PDB id: 1NBP.^17^). Based on these findings, we started our simulation studies.

**Table 1:** Crystal Structures of Human IL-2

identifier	method	positions	explanation		reference
1M47	X-ray	21–153	human IL-2 (native form, structure 1)		^16^
1M48	X-ray	21–153	human IL-2 in complex with compound 1		
1M49	X-ray	21–153	human IL-2 bound to SP-1985		
1M4A	X-ray	21–153	human IL-2 carrying a Y31C substitution, covalently modified at the Cys31 residue with (1H-indol-3-yl)-(2-mercapto-ethoxyimino)-acetic acid		
1M4B	X-ray	21–153	human IL-2 carrying a K43C substitution, covalently modified at the Cys43 residue with 2-[2-(2-cyclohexyl-2-guanidino-acetylamino)-acetylamino]-N-(3-mercapto-propyl)-propionamide		
1M4C	X-ray	21–153	human IL-2 (native form, structure 2)		
1NBP	X-ray	21–153	human IL-2 carrying a Y31C substitution, covalently modified at the Cys31 residue with 3-mercapto-1-(1,3,4,9-tetrahydro-B-carbolin-2-yl)-propan-1-one/binding sites elucidated (Phe42, Arg38, and Leu72)		^17^
1PW6	X-ray	21–153	low micromolar inhibitor targeting IL-2	the critical hot spots (binding sites; F42, E62) involved in IL-2 binding to IL-2Rα have been elucidated.	^18^
1PY2	X-ray	21–152	crystal structure of a 60 nM small organic molecule bound to a hot spot residues on IL-2		
1QVN	X-ray	21–152	structural model of SP4160 engaged with IL-2 carrying a V69A substitution		^19^
1Z92	X-ray	21–153	IL-2 bound to IL-2Rα		^20^
2B5I	X-ray	21–153	multimeric complex of IL-2 bound to IL-2Rα, IL-2Rβ, and γ_c_		^21^
2ERJ	X-ray	21–153	crystal structure of the multisubunit IL-2 receptor bound to IL-2		^22^
5M5E	X-ray	8–153	resolved structure of a IL-2 variant in complex with IL-2R (CEA-IL2v)		^23^
1ILM	model	26–153	IL-2 in complex with IL-2Rα: alternative models A and B chains		^24^
1ILN	model	26–153			
1IRL	NMR	21–153	solution structure of hIL-2 carrying a F42A substitution determined by NMR and X-ray, compared to wild-type IL-2		^25^
3INK	X-ray	21–153	structure of human recombinant IL-2 with C125A replaced		^26^
3QAZ	X-ray	21–153	IL-2 mutant D10 ternary complex		^5^
3QB1	X-ray	21–153	IL-2 mutant D10		
4NEJ	X-ray	24–153	crystal structure of human IL-2 with a small compound fragment bound at the crystal contact interface		^27^
4NEM	X-ray	24–153			
5LQB	X-ray	22–153	complex structure of mutant hIL2 (proleukin) bound to the fab fragment of NARA1 antibody		^28^
5UTZ	X-ray	21–153	human IL-2/Fab complex		^29^
6LX3	EM	1–21	Cryo-EM structure of human secretory immunoglobulin A		^30^
6LXW	EM	1–21	Cryo-EM structure of human secretory immunoglobulin A in complex with the N-terminal domain of SpsA		
6VWU	X-ray	-	X-ray structure of ALKS 4230, a circularly permuted human IL-2 fused to IL-2Rα		^31^
6YE3	X-ray	21–153	crystal structure of human IL-2 in complex with a Fab fragment from UFKA-20		^32^
7DR4	X-ray	21–153	crystal structure of human IL-2 in complex with an antihuman IL-2 antibody		^33^
7M2G	X-ray	21–153	human IL-2 variant carrying P65K and C125S substitutions		^34^
7RAA	X-ray	21–153	designed IL-2 which is called as StabIL-2 seq15		^34^
7RA9	X-ray	21–153	designed IL-2 which is called as StabIL-2 seq1		
7ZMZ	X-ray	20–153	engineered IL-2 bound to its target IL-2Rα receptor		^35^
7YZJ	X-ray	84–92	FAB complex crystallized with antigenic peptide of IL-2		^36^
AF-P60568-F1	predicted	1–153	interleukin-2 alphafold structure prediction		^37^

**Figure 2:**
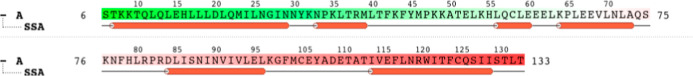
Secondary structure of the IL-2 crystal structure (PDB id: 1M47) represented in 2D. The red barrels indicate α-helices.

**Figure 3:**
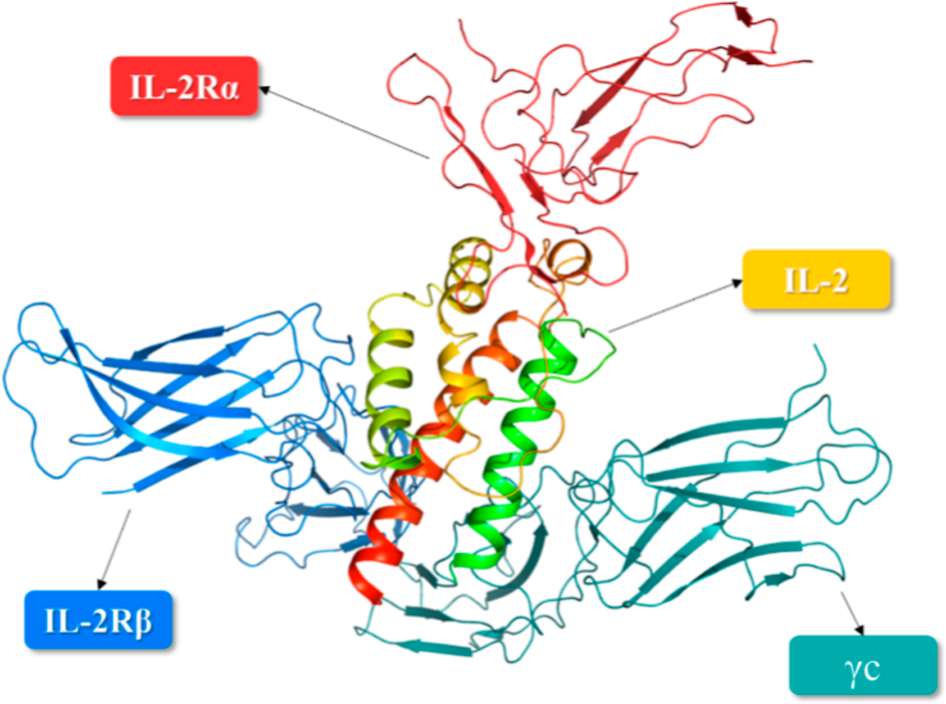
Interactions between the IL-2 and IL-2R subunits are depicted as a cartoon representation.^22^

### 2.3 MD Simulations

The formulation components of lipid-based formulations were constructed using the Maestro Interface.^38^ The atomic types and protonation forms were defined according to the OPLS4 force field. The Protein Preparation Wizard^39^ was applied to define atom parameters, add predicted positions for missing side chains, and optimize the protein structure. The chemical compounds were processed using LigPrep at pH 7.4 ± 1.0.^40^ MD simulation systems were constructed using the System Builder utility for execution in Desmond.^41^ The TIP3P model was utilized for the water molecules. The simulation system relaxation followed the software’s default protocol. The recording interval was set to 10 ps to save MD trajectories together with the calculated energy for each frame.

MD simulations are fast and economical and can provide savings in terms of time and cost by reducing the number of experimental studies. However, it will not be possible to simulate with all known excipients within the scope of the study due to the duration and size of the simulation. For this reason, simulations were carried out with the excipients commonly used for lipid-based formulations to limit the excipients. Accordingly, the excipients selected for the simulations are presented in Table 2.

**Table 2:** Excipients Were Evaluated In Silico Effects on the Formulation

long-chain fatty acids	medium-chain triglycerides	long-chain mono- and diglyceride mixtures	propylene glycol esters	water-soluble surfactants	co-surfactants
Oleic acid	Labrafac lipophile WL 1349	Maisine	Capryol 90	Labrasol	Transcutol HP
		Peceol	Lauroglycol 90		

For previous studies, Capryol 90 (propylene glycol monocaprylate), Labrafac (propylene glycol dicaprolate/dicaprate), Labrasol (caprylocaproyl polyoxyl-8 glycerides), Lauroglycol 90 (propylene glycol monolaurate), Maisine (glyceryl monolinoleate), Peceol (glyceryl monooleate), and Transcutol HP (diethylene glycol monoethyl ether) were provided by Gattefossé company (France). The molecules and their ratios in the simulations were determined based on the technical data sheets of products retrieved from the company and are summarized in Table S1. When molecular dynamics simulations with IL-2 were evaluated in terms of interaction surface and molecule size by visual inspection, a strategy was used to add 4 monomers to the simulations. Accordingly, the molecule ratios were selected based on the percentage of molecules forming the main majority, and the simulations were carried out in 4 replicates. The monomer structures are represented in Figure S1.

### 2.4 Simulation Analyses

The MD simulation analyses were conducted by using the Simulation Interaction Diagram tool of Desmond.^41^ Further analyses were applied using internally developed MD analysis scripts. The equations of the Root-Mean-Square Deviation (RMSD) and Root-Mean-Square Fluctuation (RMSF) are given in eqs 1 and 2.

1 $$RMSD\left(x,x_{ref}\right)=\sqrt{\frac{1}{n}\sum_{i=1}^{n}{\left|x_{,}-x_{i}^{ref}\right|}^{2}}$$

Equation 1. The RMSD equation that is applied on the IL-2 backbone, where *x*_*i*_ represents the backbone atomic coordinates in the conformation of interest, *x*_*i*_^ref^ represents the backbone atomic coordinates in the reference conformation, and *n* represents the total number of backbone atoms analyzed.

2 $${RMSF}_{i}=\sqrt{\frac{1}{T}\sum_{t=1}^{T}{\left(x_{,}\left(t\right)-\left\langle x_{,}\right\rangle \right)}^{2}}$$

Equation 2. The RMSF_*i*_ equation that is applied on the IL-2 residues, where the fluctuation of the *i* ^th^ residue is given by using *x*_,_(*t*) the position of the residue and ⟨*x*_,_⟩ the average position of the residue observed for a *T* number of steps of simulation.

### 2.5 In Vitro Evaluations Based on In Silico Data

Based on the in silico screening results, a multiphase water-in-oil-in-water-type SNEDDS-derived nanoemulsion system was designed with the Labrafac Lipophile WL 1349 excipient. In this system, the aqueous IL-2 solution constituted the internal phase of the primary emulsion, allowing the effective incorporation and stabilization of the hydrophilic cytokine. The self-emulsifying behavior of the formulation also facilitates dispersion upon dilution. A self-emulsifying system with Labrafac was loaded with IL-2, and the system was characterized. Labrafac was weighed, and PGPR was added (oil phase). Then, the oil and water phases were brought to the same temperature (50 °C) and mixed at 1000 rpm for 3 min. Then, it was mixed at 24,000 rpm in the ultraturrax for 3 min. It was passed through the Microfluidizer (LV1, Microfluidics, USA) at 10,000 psi. Kolliphor EL was added to the prepared primary emulsion and mixed at room temperature conditions until homogenized at 100 rpm.^42^ In the preparation of the IL-2-loaded formulation, unlike the preparation of the blank formulation, the IL-2 solution is used as the water phase in the primary emulsion.

The droplet size, polydispersity index (PDI), and zeta potential of the nanoemulsion formulations were analyzed by using a Malvern ZetaSizer (Malvern Zetasizer Nano ZS, Malvern, UK) at 25 ± 0.5 °C. For measurement, blank and IL-2-loaded formulations were diluted with 250 mL of distilled water and stirred with a magnetic stirrer at 25 °C.

Electrical conductivity measurements provide a simple and inexpensive method for characterizing nanoemulsion systems. The conductivity value identifies the type of continuous phase of nanoemulsion.^43^ For formulations, conductivity tests were performed using a digital conductivity meter (Mettler Toledo Seven2Go Cond meter) for samples immediately after preparation. The conductivity values were measured at room temperature and were recorded in μS/cm.

Surface tension measurements for the prepared formulations were carried out using the pendant drop method with an Attension Theta Lite instrument (Biolin Scientific, Finland). The experiment was carried out at room temperature. The measurements are calculated by using the Young Laplace equation.^44^

Transmission electron microscopy (TEM, FEI Technai G2 Spirit BioTwin, USA) was employed to investigate the morphological properties of the formulations. Formulations were diluted 1:250 with distilled water, and both the diluted formulations and a 1% w/v phosphotungstic acid solution were dropped onto a copper grid.

Heating–cooling test and freezing–thawing test were performed to evaluate the thermodynamic stability of the formulations. For the heating–cooling test, the formulations were tested for three cycles, each at a temperature between 4 and 40 °C, for no less than 48 h. For the freezing–thawing test, the formulations were exposed to temperatures between −20 and 25 °C for three cycles, each at a time no less than 48 h.^45^ As a result of this cycle, phase separation was checked, and the durability of the formulations was evaluated. In addition, the droplet size, PDI, and zeta potential of formulations were analyzed after diluting with 250 mL of water.

### 2.6 Cell Culture Studies

Cell culture studies were conducted using the A498 human renal carcinoma cell line and human peripheral blood mononuclear cells (PBMCs). A498 cells were maintained in Eagle’s Modified Eagle Medium (EMEM) supplemented with 10% FBS and 1% penicillin/streptomycin, while PBMCs were cultured in RPMI-1640 medium containing the same supplements. All cells were cultured at 37 °C in a humidified incubator with 5% CO_2_. Cells incubated with the medium alone served as the control group.

The biological activity of IL-2 in nanoemulsion formulations was assessed through a proliferation assay performed on PBMCs to determine whether the activity of IL-2 was preserved during nanoemulsion preparation. PBMCs were seeded into U-bottom 96-well plates (2 × 10^4^ cells/well), and 5 ng PMA was added to each well to stimulate PBMC cells. After 24 h of incubation at 37 °C and 5% CO_2_, serial dilutions of IL-2-loaded nanoemulsions, free IL-2 solutions, and blank formulations (ranging from 0.01 nM to 10 μM) were added. Following 48 h of incubation, PBMC proliferation and viability were evaluated using the WST-1 assay by measuring absorbance at 450 nm using a microplate reader^46^ (SpectraMax iD3, Molecular Devices). The WST-1 assay measures cellular metabolic activity based on the reduction of tetrazolium salt by mitochondrial dehydrogenases in metabolically active cells, providing an indirect indicator of viable and proliferating cells.

The anticancer activity of the IL-2-loaded nanoemulsion and free IL-2 solution was evaluated using a PBMC and A498 coculture model. In coculture experiments, PBMC cells are employed as effector cells and A498 cells as target cells. PBMCs were stimulated by incubating them with blank formulation, IL-2-containing nanoemulsion formulations, or IL-2 solution at the same ratio for 48 h. A498 cells were seeded at a density of 1 × 10^6^ cells/mL in 96-well U-bottom cell culture plates and allowed to attach for 24 h. Stimulated PBMCs were then cocultured with A498 cells at A498/PBMC ratios of 1:1, 1:5, and 1:10. After 24 h of coculture, cell viability was assessed using the WST-1 assay.^46^ Unstimulated PBMC-added cultures and blank-formulation-treated groups were included as control conditions.

In addition, to better mimic the tumor microenvironment, anticancer activity was evaluated in a 3D coculture model using A498 spheroids embedded in growth factor-reduced Matrigel (Corning, cat. No. 354234, product code 11543550, Lot no: 29024002). This Matrigel formulation is enriched in extracellular matrix components, including laminin, collagen IV, and entactin, while containing reduced levels of endogenous growth factors. The use of growth factor reduced Matrigel-minimized matrix-derived proliferative signaling, thereby enabling a more accurate evaluation of immune cell-mediated cytotoxic effects in the 3D coculture model.^47^ Plates were first coated with poly-HEMA to generate nonadherent surfaces. A498 cells were seeded at a density of 5 × 10^3^ cells/well in medium containing 3% (v/v) Matrigel and centrifuged at 1000 rpm for 10 min to promote spheroid formation. Spheroid development was monitored microscopically, and mature spheroids were obtained after 7 days. PBMCs prestimulated with IL-2-loaded nanoemulsion, free IL-2 solution, or blank formulation were added to spheroids at A498/PBMC ratios of 1:1, 1:5, and 1:10. After 48 h of coculture, cell viability was assessed using the WST-1 assay, and results were expressed as relative metabolic activity compared with control groups. Spheroids incubated with the medium alone served as the negative control. In parallel, cytotoxicity was assessed in separate plates using a commercial LDH assay kit.^48^ The release of LDH was measured in the control group, which was incubated only in the medium. The positive control group was treated with 1% Triton X-100, which is a recognized cytotoxic agent.

### 2.7 Statistical Analysis

All quantitative data are presented as mean ± standard deviation (SD). Cell culture experiments were analyzed using two-way analysis of variance (two-way ANOVA) to evaluate the effects of IL-2 solution vs IL-2-loaded nanoemulsion and cell ratio/concentration, followed by Tukey’s multiple comparisons test. A p value <0.05 was considered statistically significant. Statistical analyses were performed using GraphPad Prism 9.0.0.

## 3 Results and Discussion

### 3.1 Analysis of IL-2 Binding Sites on IL-2 Receptors: Surface Amino Acids and Bond Types

The IL-2/IL-2R binding sites were examined using the 2ERJ structure (Figure 3). In this analysis, the relevant surface amino acids involved in the interaction and the types of bonds formed between these amino acids and IL-2 receptors were determined. The results, including the detailed list of relevant surface amino acids and the corresponding bond types, are comprehensively presented in Figure 4 and Table S2. As previously reported, the IL-2 binding site residues interact with IL-2Rα (Arg38, Phe42, Glu62, and Leu72), which were highlighted as relevant surface amino acids.^17^

**Figure 4:**
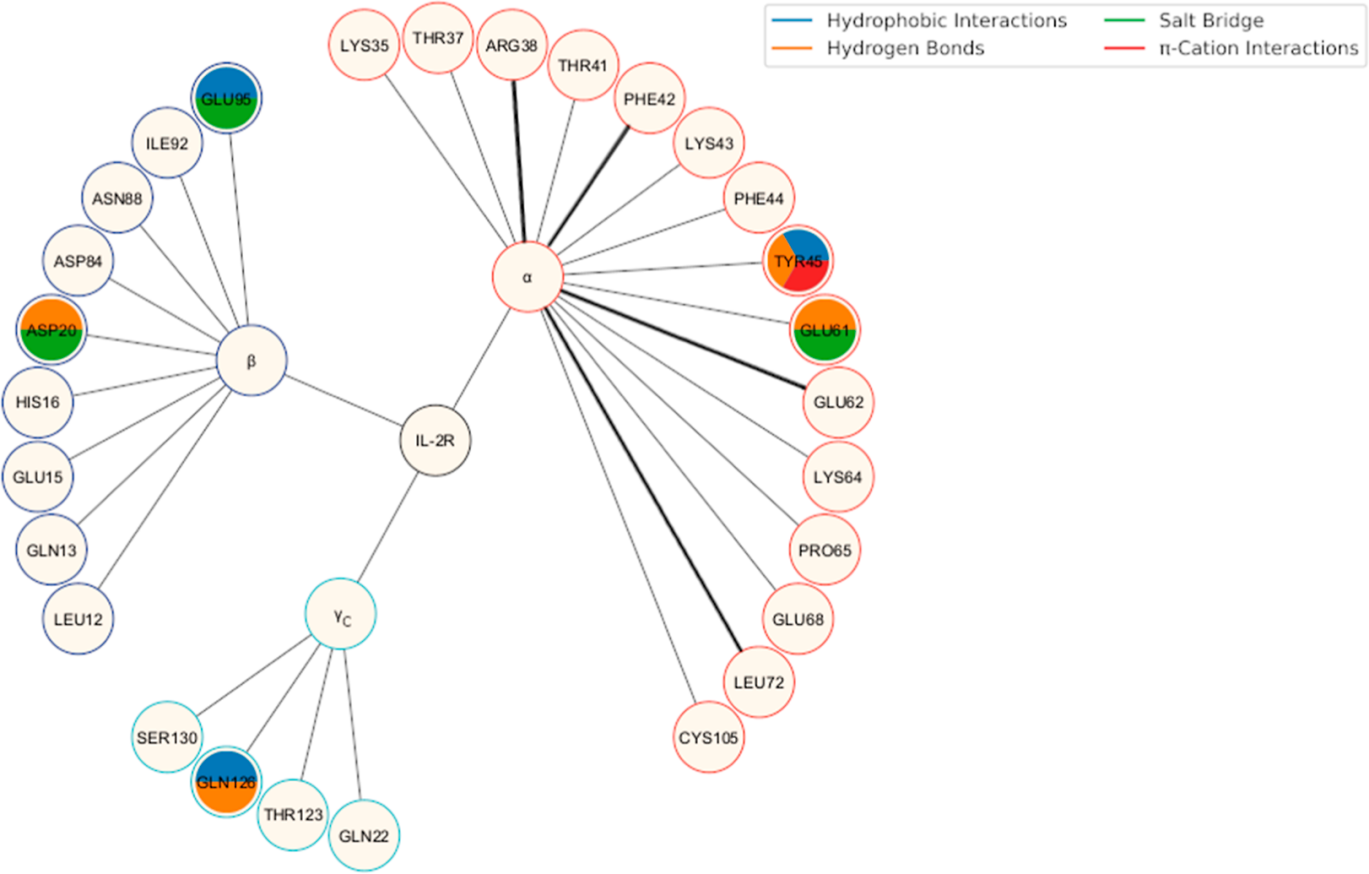
Binding site residues of IL-2 interacting with IL-2R are represented as nodes and colored based on the observed interaction types and domains. The key IL-2 residues involved in interactions with IL-2Rα are highlighted as bold lines. The α, β, and γ_c_ subunits of IL-2R are presented in red, blue, and cyan circles, respectively.

### 3.2 Performing MD Simulations and Interaction Analyses with Excipients

Lipid-based drug delivery systems are among the promising formulation approaches for therapeutic peptides and proteins planned to be administered orally. These delivery systems are designed to overcome epithelial, mucus, and enzymatic barriers, which ultimately enable access to the systemic circulation. Thus, lipid-based systems can increase the bioavailability of drugs and provide a protective environment to preserve the effectiveness of peptides and proteins.^49^ The advantage of the conducted simulation study is that it offers insights into the molecular interactions between the active pharmaceutical ingredient and excipients prior to the formulation experiments. Because of that, MD simulations were performed to determine the appropriate excipients by observing the interactions of IL-2Rα binding sites and excipients with MD simulations for the anticancer effect.

The excipients are a kind of mixture, and these excipients were drawn based on their mixture compositions and percentages. The excipients selected for use in MD simulation are presented in Table 2. Reversible interactions between interleukin-2 and monomers were observed over 100 ns. The snapshots were taken at 0, 33.3, 66.6, and 100 ns. The snapshots of the simulations with these excipients are presented in Figure 5.

**Figure 5:**
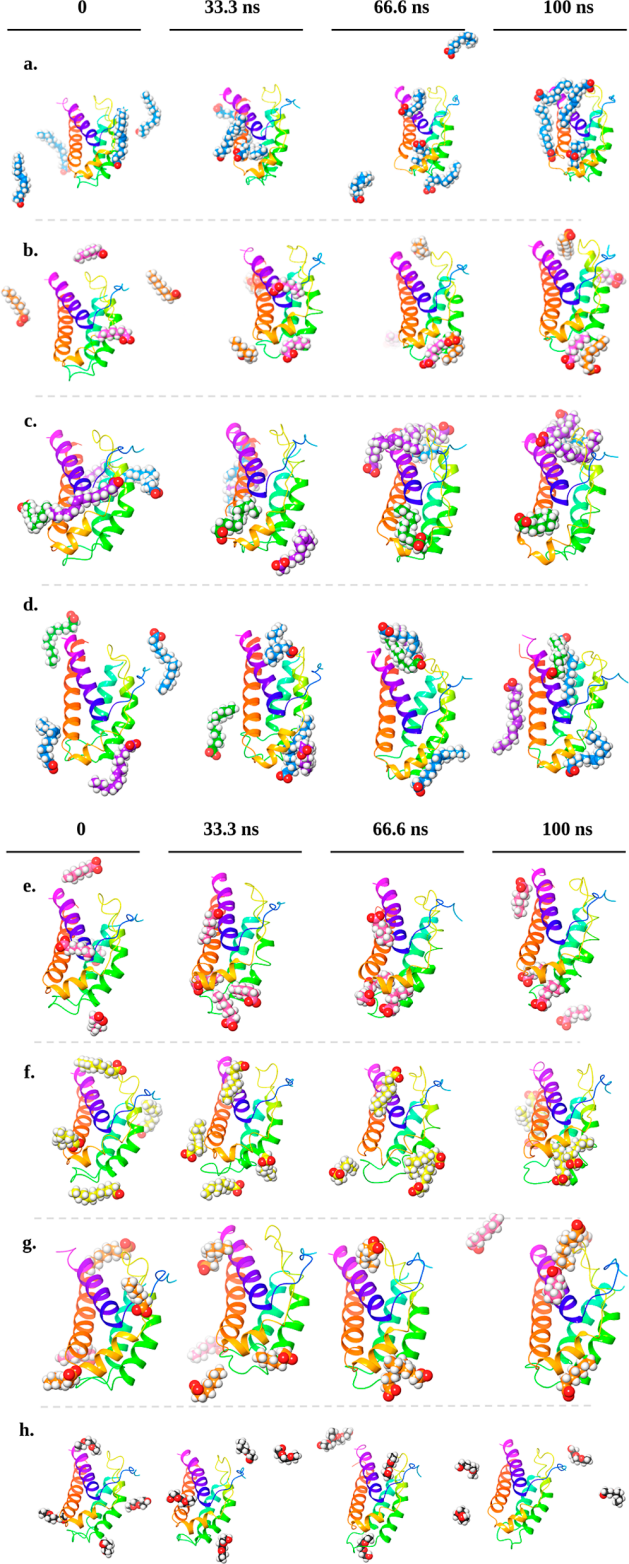
Snapshots of the IL-2’s simulations with these excipients. (a) Oleic acid (the carbon atoms of oleic acid molecules were presented in blue). (b) Labrafac Lipophile WL 1349 (the carbon atoms of capric acid and caprylic acid molecules were presented in orange and pink, respectively). (c) Maisine (the carbon atoms of oleic acid, palmitic acid, and linoleic acid molecules were presented in blue, green, and purple, respectively). (d) Peceol (the carbon atoms of palmitic acid, linoleic acid, and oleic acid molecules were presented in green, purple, and blue, respectively). (e) Capryol 90 (the carbon atoms of caprylic acid molecules were presented in pink). (f) Lauroglycol 90 (the carbon atoms of lauric acid molecules were presented in yellow). (g) Labrasol (the carbon atoms of capric acid and caprylic acid molecules were presented in orange and pink, respectively). (h) Transcutol HP (the carbon atoms of diethylene glycol monoethyl ether molecules were presented in gray).

When examining the snapshots in Figure 5, it is observed that the monomers do not adhere to the structure and form reversible interactions. Conversely, in simulations conducted with Transcutol HP, the monomers maintain a distance from the IL-2 molecule, which is related to a low interaction between the molecules.

The interactions observed as a result of simulations performed with 4 replicates are summarized in Figure 6. According to the IL-2 and receptor interaction regions presented in Table S3, the interaction regions were matched with the subunits of IL-2R (see also Figure S2). According to the graphs, the monomers and IL-2 molecules make different types of interactions, such as hydrophilic and hydrophobic contacts, with different amino acids in 100 ns.

**Figure 6:**
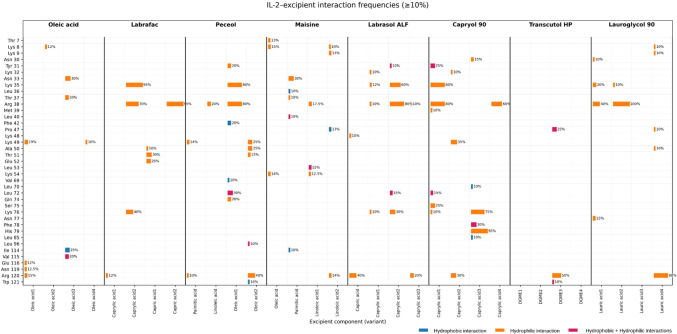
Graphical representation of the interaction analysis between IL-2 and excipients.

Generally, reversible interactions between IL-2 and the monomers were observed. The oleic acids make hydrophilic and hydrophobic contacts with different amino acids. Maisine, consisting of oleic, palmitic, and linoleic acids, interacted with Arg 38, Phe 42, and Leu 72 of its binding sites to IL-2Rα. Significant long-term contacts with IL- 2Rα binding sites were established in repeated simulations. Peceol, which contains different ratios of oleic, palmitic, and linoleic acids, exhibited high interactions with IL-2Rα binding sites like Maisine. Specifically, Peceol’s linoleic acid and oleic acid molecules established hydrophilic contact with Arg 38 and hydrophobic contact with Phe 42. Capryol 90, containing caprylic acid molecules, and hydrophilic contacts were consistently established with Arg 38, while hydrophobic contacts with Phe 42 were limited to less than 15 ns. Repeated simulations showed hydrophilic and hydrophobic contacts with Leu 72 lasting 15 to 60 ns since it interacts with the surface amino acids of Arg 38, Phe 42, and Leu 72. Lauroglycol 90, composed of lauric acid molecules, formed irreversible hydrophilic contacts with Arg 38. Two different lauric acid molecules within the simulation system primarily mediated these contacts. Labrasol formed the hydrophilic contacts established with Arg 38 that continued throughout the simulation. This result was found to be reproducible in the study carried out with four replications. Transcutol HP, consisting of diethylene glycol monoethyl ether molecules, demonstrated limited interaction (less than 10%) with surface amino acids that are important for the antitumor effect. While hydrophilic and hydrophobic contacts with various amino acids were limited, no incompatibilities were expected.

Labrafac Lipophile WL 1349 consists of capric and caprylic acid molecules and showed high occupancy values (95% and 70%, respectively) for the key residue Arg 38 and also reversible interactions (below 100%, without poisoning the protein). Since the interaction with Arg 38 takes a long time, its binding to IL-2Rα might be prevented, and thus, a superior antitumor effect might be achieved in comparison to its existing solution form.

RMSF analyses also showed that the systems that do not show high occupancy values between the excipient components and IL-2 (Labrasol and Transcutol) had less fluctuation in the IL-2 residues during the simulations. The remaining systems where IL-2’s interaction resulted more fluctuations during simulations (Figure 7) without affecting the general structure as can be seen in RMSD plots (Figure 8). It is understood that the structures do not undergo a significant conformational change since their RMSD values are below 3. The inference was also supported by a visual analysis. Although the RMSD value slightly exceeded 2 Å in the simulations of two copies of Maisine, two copies of Peceol, one copy of oleic acid, and one copy of Labrafac, it is not expected that the IL-2 structures will undergo significant conformational changes or denaturation due to these minor variations. Based on the in silico findings given above, the formulation studies were decided to be carried out with Labrafac Lipophile WL 1349.

**Figure 7:**
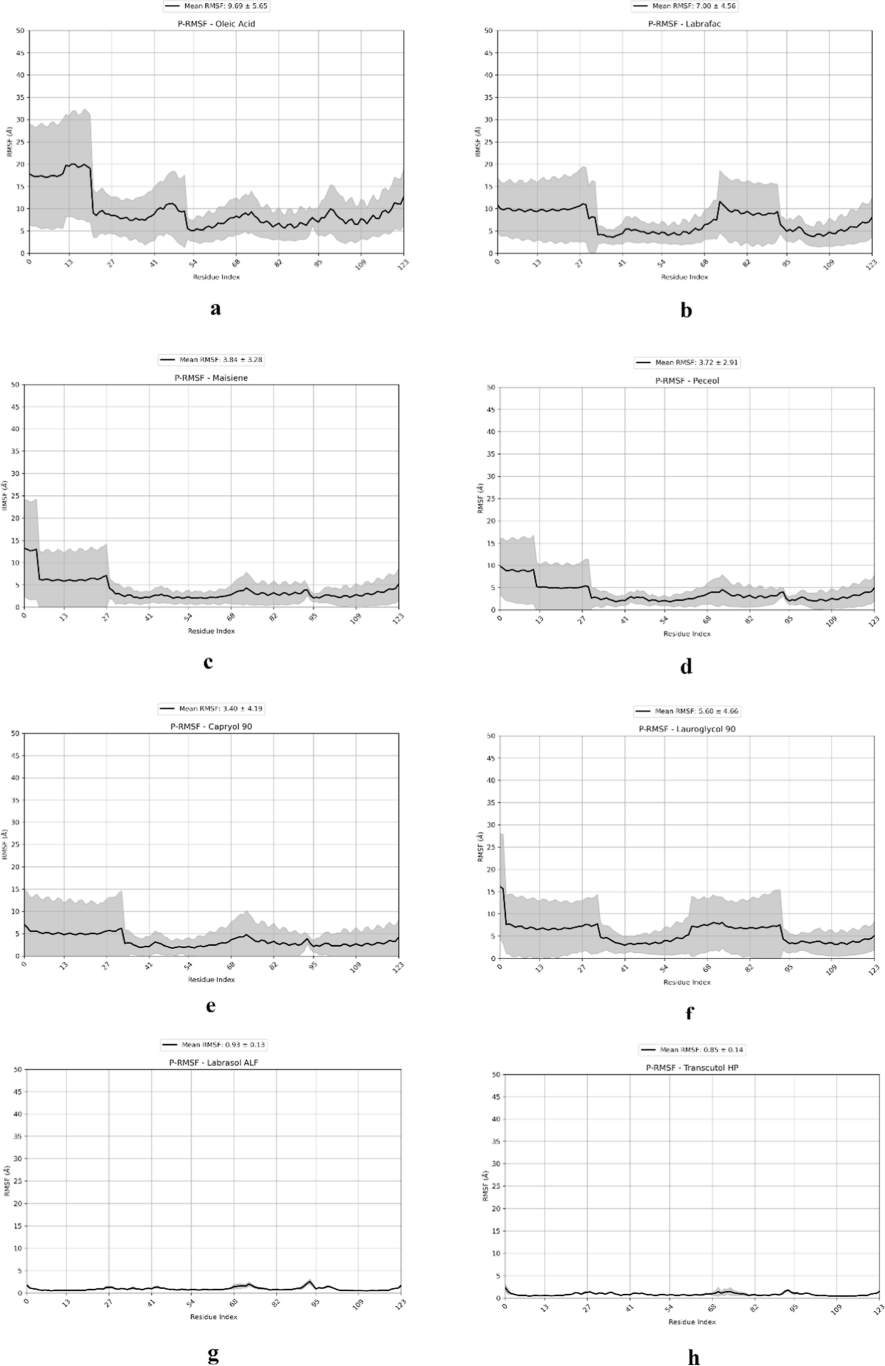
RMSF graph shows the flexibility of different residues of the IL-2 structure during the MD simulations in the presence of (a) Oleic acid, (b) Labrafac Lipophile WL 1349, (c) Maisine, (d) Peceol, (e) Capryol 90, (f) Lauroglycol 90, (g) Labrasol, and (h) Transcutol HP. The average RMSF value of each residue was reported in black, and standard deviations were represented as gray zones for each residue of IL-2.

**Figure 8:**
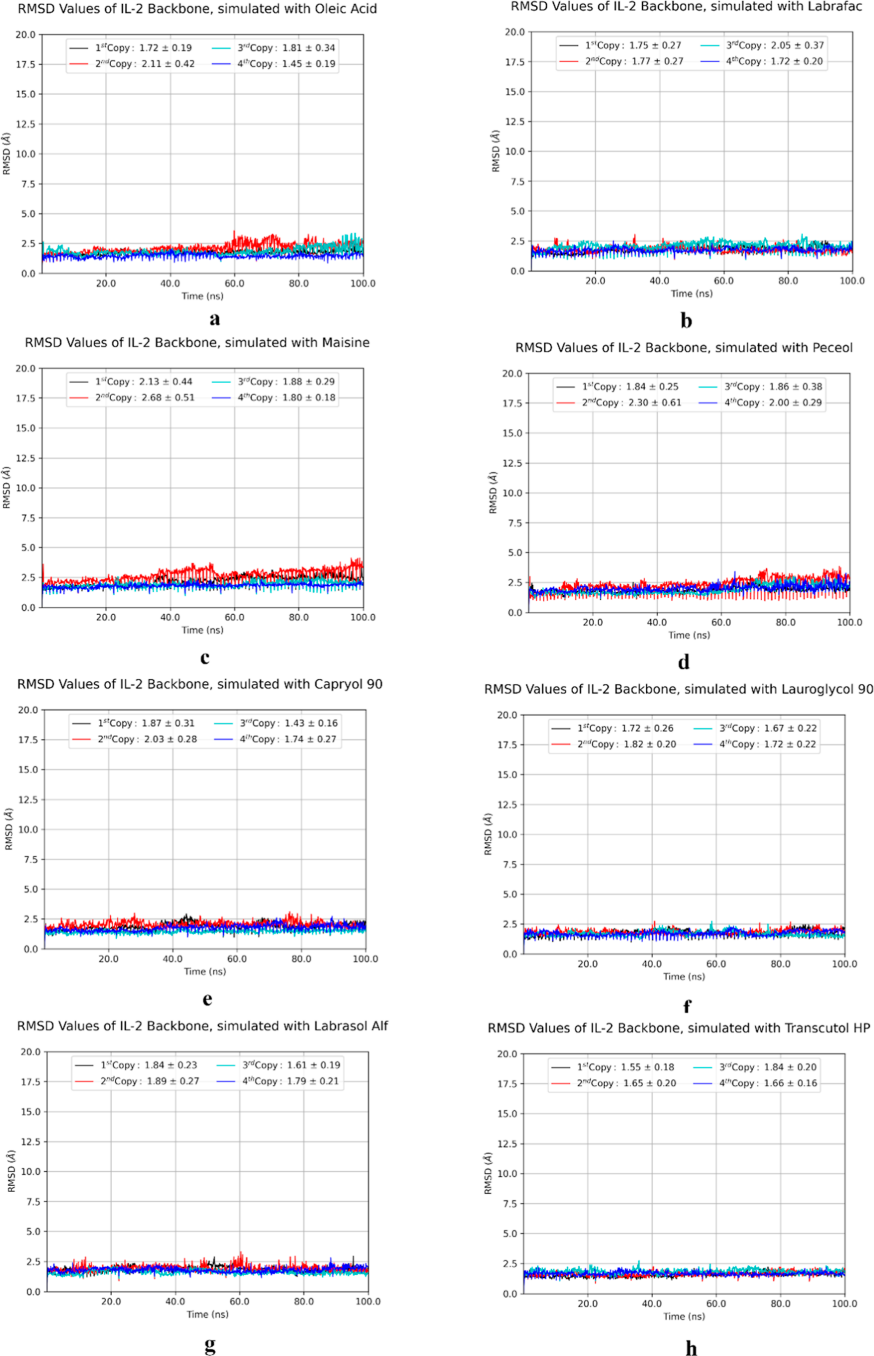
RMSD graph shows the similarity of the IL-2 structure in the presence of (a) Oleic acid, (b) Labrafac Lipophile WL 1349, (c) Maisine, (d) Peceol, (e) Capryol 90, (f) Lauroglycol 90, (g) Labrasol, and (h) Transcutol HP to the state when the system was first started during the simulation process. Each copy is represented in a different color.

In silico prescreening offers a significant advantage in formulation studies involving recombinant proteins, as it reduces experimental workload and minimizes protein-related costs. The MD-based approach employed in this study offers an efficient strategy for narrowing down excipient candidates prior to laboratory studies, thereby enabling a more rational and cost-effective formulation development. MD simulations were employed to provide a molecular-level understanding of IL-2-excipient interactions within the formulation at the preadministration stage. Following oral administration, partial or complete dissociation and reorganization of lipid excipients are expected due to physiological dilution and digestion processes. Therefore, the MD results in this study do not directly simulate in vivo behavior but rather evaluate formulation stability and excipient–protein interactions.

### 3.3 In Vitro Evaluations Based on In Silico Studies

The characterization results of IL-2-loaded and blank formulations are presented in Table 3, while the thermodynamic stability results obtained after each cycle are shown in Figure 9. TEM images of IL-2-loaded and blank formulations are shown in Figure 10, and TEM images illustrated the formation of spherical shapes.

**Table 3:** Characterization and Thermodynamic Stability Results of Formulation^a^

characterization results		
	blank formulation	IL-2-loaded formulation
droplet size (nm)	96.89 ± 1.70	103.90 ± 2.33
PDI	0.181 ± 0.002	0.213 ± 0.009
zeta potential (mV)	–8.04 ± 0.90	–5.17 ± 0.93
conductivity (μS/cm)	34.60 ± 0.92	23.00 ± 0.03
surface tension (mN/m)	28.92 ± 0.11	29.26 ± 0.21

a Independent variables results are given as mean ± SD, *n* = 3.

**Figure 9:**
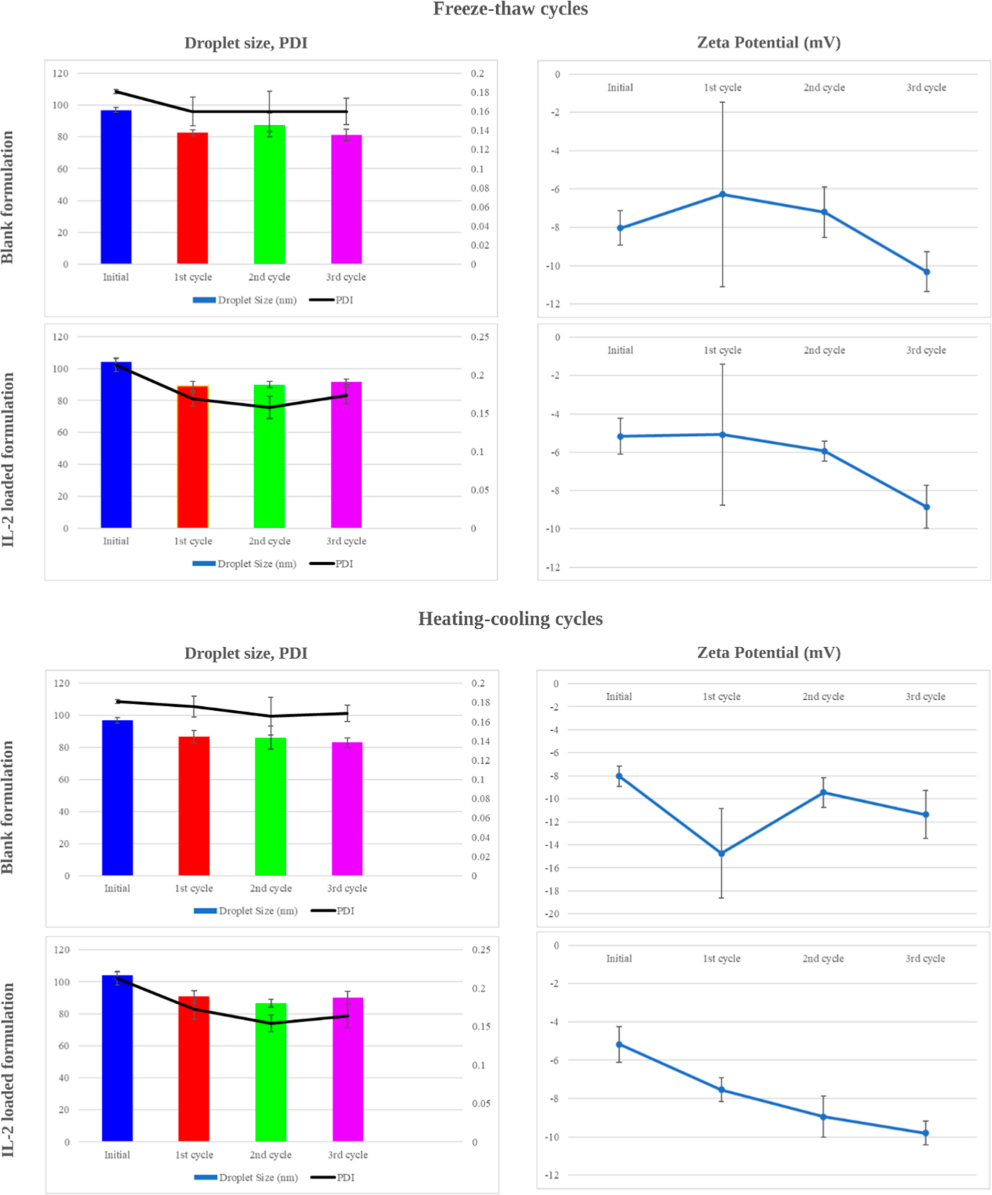
Droplet size, polydispersity index (PDI), and zeta potential of the formulations before and after freezing–thawing and heating–cooling cycles.

**Figure 10:**
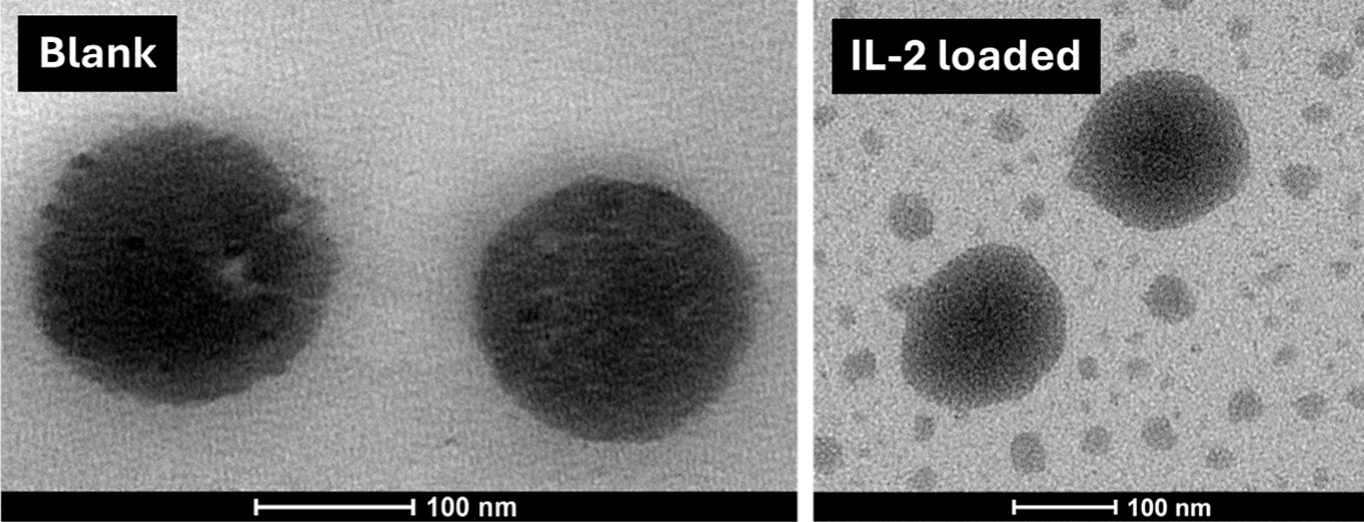
TEM images of formulations.

The evaluated characteristics of the IL-2-loaded and blank systems, including droplet size, PDI, zeta potential, conductivity, surface tension, thermodynamic stability, and TEM images, were within the targeted range. The IL-2 loading caused a slight increase in the droplet size of the nanoemulsion system. This suggests that IL-2 may influence the structural properties of the emulsion and potentially impact its stability. The PDI values (∼0.2) indicated a homogeneous, monodisperse distribution. Additionally, IL-2 may affect the surface charge of the droplets, possibly reducing the ionic strength and electrical conductivity in the system. In conclusion, while IL-2 loading has caused changes in some of the physicochemical properties of the nanoemulsion, these changes are generally small and stable.

The solubility and interactions of lipids or other components in the formulation systems may change, depending on temperature variations. Additionally, temperature changes may cause phase separation. All of these can directly affect the stability and efficacy of the system. Thermodynamic stability studies were conducted to evaluate these properties and assess whether the developed system is stable. Both freezing–thawing and heating–cooling cycles generally resulted in stable outcomes for the formulations. The droplet size remained largely consistent throughout the cycles, and the PDI values showed no significant changes. These results suggest that the formulation is resistant to both freezing–thawing and heating–cooling cycles with its stability remaining well preserved.

TEM images confirmed the spherical morphology and supported the observed droplet size and PDI data. IL-2 loading slightly affected the size and uniformity of the nanoemulsion system, as evidenced by the increased presence of smaller particles and a slight size reduction in the IL-2-loaded sample. Despite these changes, the spherical morphology and overall integrity of the droplets were preserved, indicating compatibility with IL-2 within the nanoemulsion system. Additionally, these physicochemical findings are consistent with the in silico predictions, supporting the idea that the selected lipid excipients interact reversibly with IL-2 without disrupting its structural stability.

### 3.4 Cell Culture Studies

The biological activity of IL-2 was assessed through its proliferative effect on PBMCs. After 48 h of incubation, the biological activity of IL-2 was evaluated based on the rate of cell increase using the WST-1 test, as shown in Figure 11. The nanoemulsion formulation and free IL-2 solutions showed no significant difference in PBMC proliferation rates over 48 h (*p* > 0.05). The proliferation assay showed that IL-2 retained its biological activity after being incorporated into the nanoemulsion formulation. At 89.5 nM, the cell growth rates were 1.40 ± 0.32 for the IL-2-loaded formulation, 1.10 ± 0.03 for the free IL-2 solution, and 0.313 ± 0.072 for the blank formulation.

**Figure 11:**
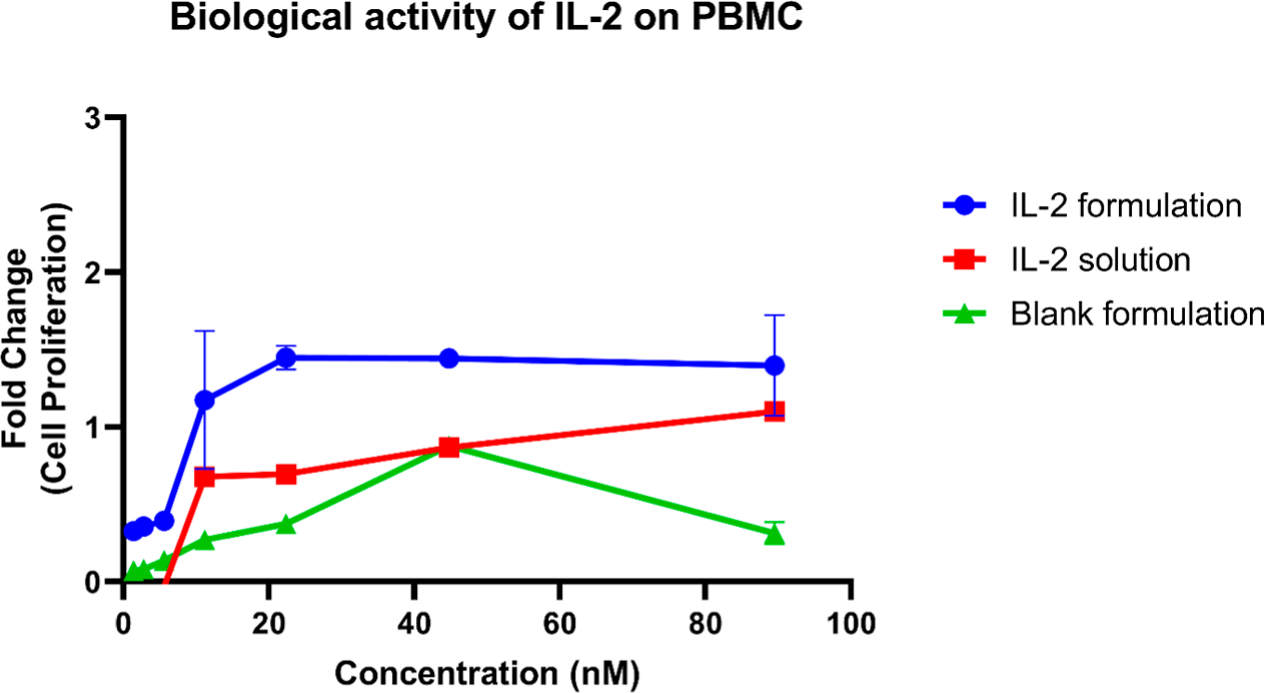
Proliferative effect of blank formulation, IL-2-loaded formulation, and free IL-2 solution on PBMCs. The blank nanoemulsion does not contain IL-2 and was tested at IL-2-equivalent dilutions corresponding to the IL-2 concentrations used in the formulation and solution groups. (Mean ± SD, *n* = 4).

The anticancer activity of the IL-2-loaded formulation and free IL-2 solution was determined using the A498/PBMC coculture model. PBMC cells were added to A498 cells at 3 different ratios, incubated for 24 h, and then tested with the WST-1 test. The coculture experiments showed that IL-2-loaded nanoemulsion enhanced the cytotoxic activity of PBMCs against A498 renal carcinoma cells more effectively than free IL-2 solutions. Figures 12 and 13 show the results for the cell viability percentage. In particular, after 24 h, at an IL-2 concentration of 89.5 nM and a PBMC/A498 ratio of 1:1, cell viability was 19.81% ± 0.51%, 60.52% ± 1.67%, and 89.1% ± 6.2% for the IL-2-loaded formulation, free IL-2 solution, and blank formulation (tested at an IL-2-equivalent dilution), respectively. This indicates that IL-2-loaded nanoemulsion significantly reduced A498 cell viability more than free IL-2 solution (*p* < 0.0001). This reduction in viability reflects enhanced PBMC-mediated cytotoxic activity against A498 tumor cells induced by IL-2, rather than a direct cytotoxic effect of the formulation. In 3D cell culture studies, the A498 spheroid formed after 7 days of development as shown in Figure 14. In the 3D spheroid model, IL-2-loaded nanoemulsions again exhibited greater PBMC-mediated cytotoxicity than free IL-2, particularly at higher PBMC/A498 ratios, as reflected by the WST-1-derived viability values (Figure 12). In the 3D spheroid model, at the same IL-2-equivalent dilution corresponding to 89.5 nM and a PBMC/A498 ratio of 1:1, cell viability was 22.61% ± 0.83%, 41.6% ± 1.2%, and 98.1% ± 5.5% for the IL-2-loaded formulation, free IL-2 solution, and blank formulation, respectively. Additionally, cytotoxicity was determined based on the measured LDH activity, as illustrated in Figure 13. LDH assays revealed considerably increased levels of cell death in spheroids treated with IL-2-loaded nanoemulsions. Overall, these findings indicate that IL-2 retains its biological activity after loading into the nanoemulsion and that the formulation enhances PBMC-mediated anticancer efficacy against A498 renal carcinoma cells in both 2D and 3D coculture models.

**Figure 12:**
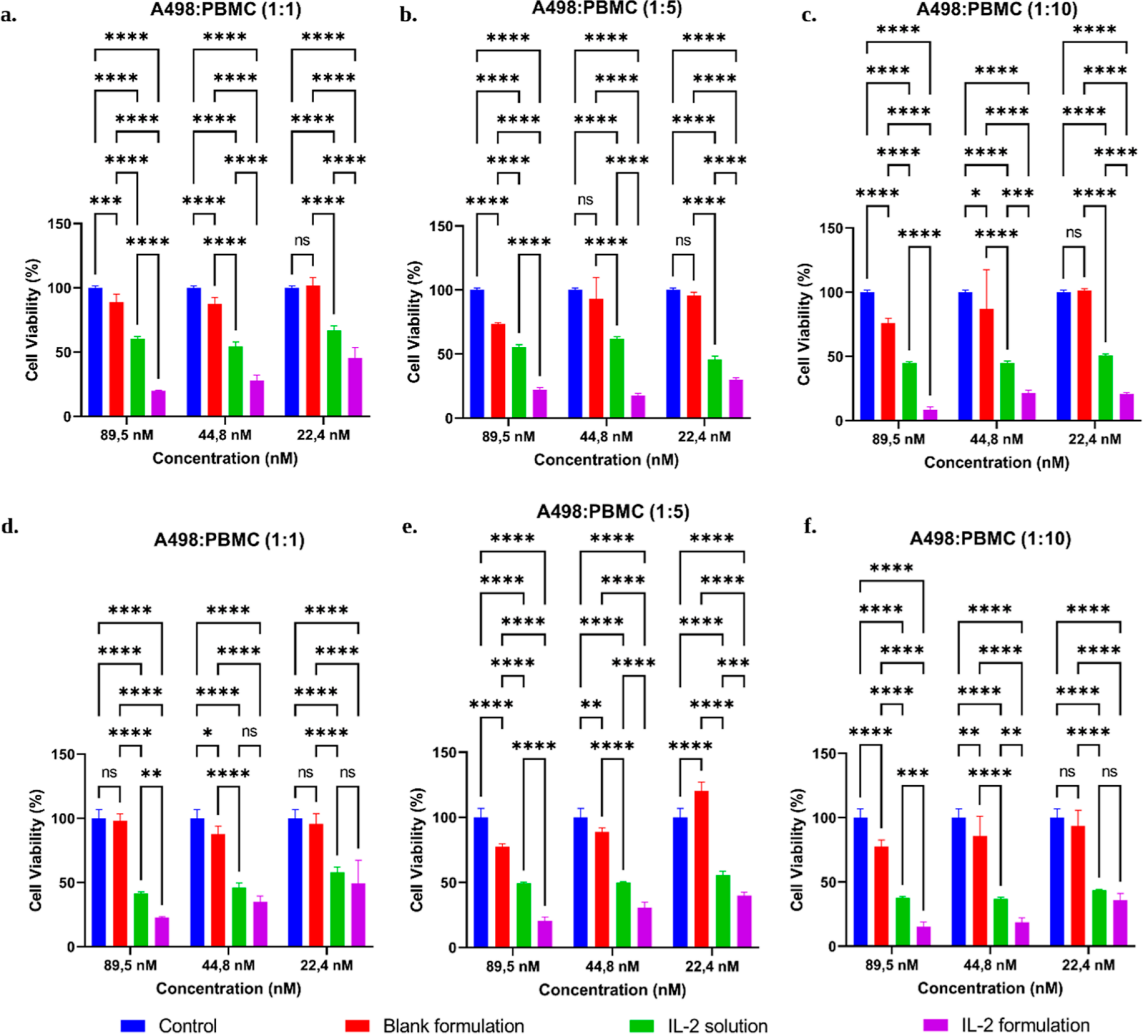
Cell viability graphs of cell culture studies with coculture of A498 and PBMC cells. Cell viability: (a–c) Conventional 2D cell culture studies at A498/PBMC ratios of (a) 1:1, (b) 1:5, and (c) 1:10. Cell viability: (d–f) 3D cell culture studies at A498/PBMC ratios of (d) 1:1, (e) 1:5, and (f) 1:10. Blank nanoemulsion does not contain IL-2 and was tested at IL-2-equivalent dilutions to ensure equivalent excipient concentrations (Mean ± SD, *n* = 4). *: *p* < 0.05; **: *p* < 0.005; ***: *p* < 0.0005; and ****: *p* < 0.0001.

**Figure 13:**
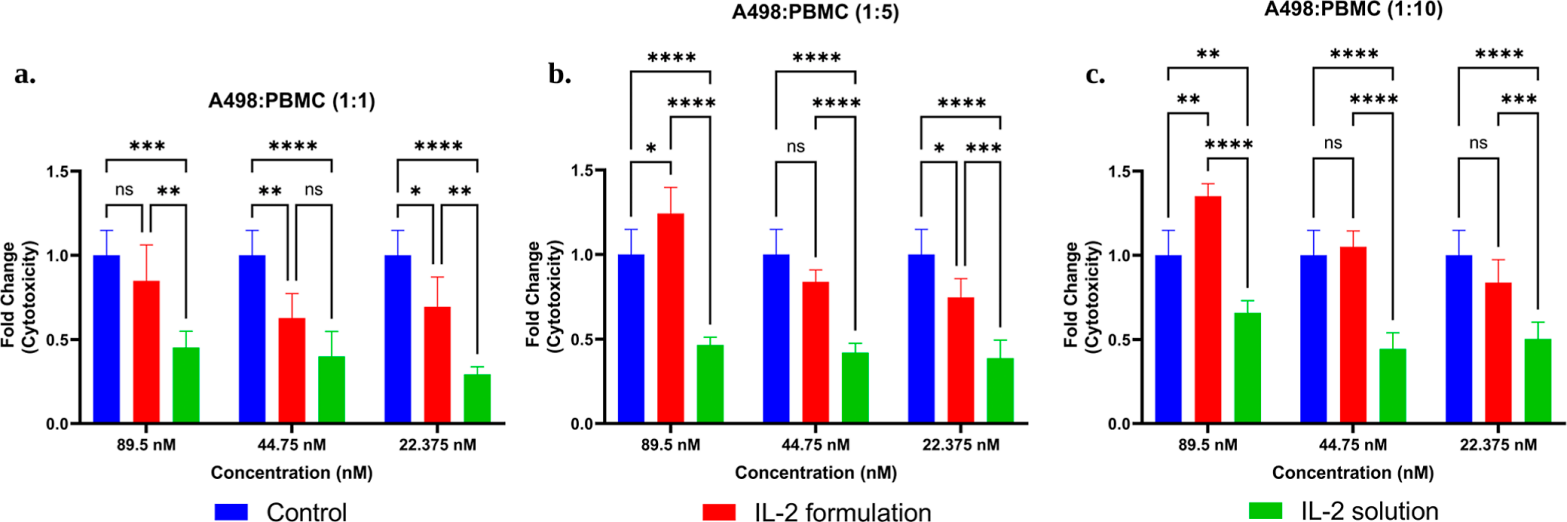
Cytotoxicity graphs of cell culture studies with coculture of A498 and PBMC cells assessed by LDH release. A498/PBMC ratios of (a) 1:1, (b) 1:5, and (c) 1:10. (Mean ± SD, *n* = 4). *: *p* < 0.05; **: *p* < 0.005; ***: *p* < 0.0005; and ****: *p* < 0.0001.

**Figure 14:**
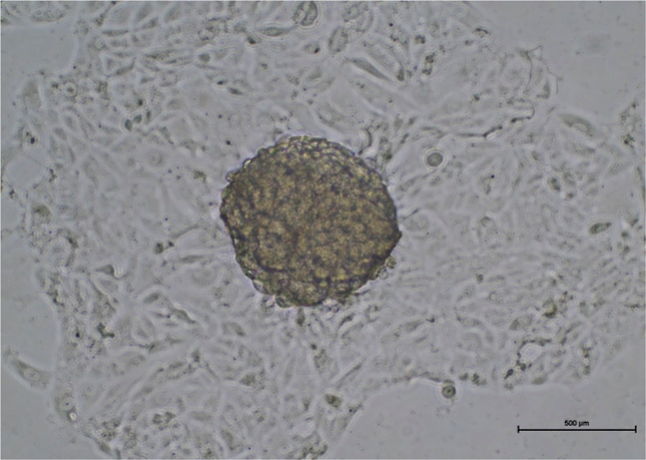
Microscopic image of the 3D spheroid tumor formed with A498 human renal carcinoma cells. There was only one spheroid in each well (scale bar: 500 μm). The image was acquired prior to PBMC addition and represents tumor globules formed under nonadherent conditions.

The enhanced anticancer activity observed for the IL-2-loaded nanoemulsion can be interpreted in the context of the molecular interaction profiles obtained from the MD simulations. These simulations indicate that selected excipients are capable of forming transient and reversible interactions with IL-2 surface residues involved in IL-2Rα binding. Such interactions may persist temporarily following the release of IL-2 from the nanoemulsion droplets, potentially influencing early receptor engagement. In parallel, nanoemulsion-based encapsulation is expected to improve local availability and cellular exposure of IL-2, which may contribute to the increased PBMC-mediated cytotoxicity observed in both 2D and 3D coculture models, while in the present study, the consistency between in silico interaction trends and in vitro biological outcomes supports a formulation-driven contribution to the observed enhancement in anticancer efficacy.

## 4 Conclusions

This study demonstrated that molecular-dynamics-based screening can effectively guide excipient selection for IL-2 nanoemulsion formulations by revealing key reversible interactions between IL-2 and lipid excipients. Labrafac Lipophile WL 1349 showed the most favorable interaction profile, providing prolonged and reversible contacts with Arg38 and other IL-2Rα-associated residues, which may suggest partial shielding of the α-subunit binding interface. These observations indicate a potential for modulation of IL-2Rα engagement while maintaining the protein’s structural integrity within the lipid environment.

The optimized nanoemulsion maintained IL-2 biological activity and produced significantly enhanced anticancer efficacy against A498 renal carcinoma cells compared with the IL-2 solution in PBMC coculture models. In this coculture setting, the observed effects are consistent with immune effector-mediated tumor cell killing rather than nonspecific cytotoxicity. The tumor cell-to-PBMC ratio is an important experimental variable in immune–tumor coculture models, as it directly influences the magnitude of immune effector activation and tumor cell killing. In the present study, evaluation across multiple tumor cell-to-PBMC ratios enabled a more robust assessment of formulation-induced immune effects. These findings underscore the importance of considering tumor cell to PBMC ratios when interpreting in vitro immuno-oncology data and support the inclusion of ratio-dependent analyses to improve the translational relevance of future studies.

Overall, these findings support that integrating in silico screening with formulation development can rationally reduce the need for experimental studies and associated costs while enabling the design of excipient–protein combinations that support targeted biological outcomes. Within the scope of this study, this strategy may represent a promising approach for the development of immunotherapeutic formulations for IL-2.

## Supplementary Material



## Notes

Contents of excipients; molecular structures of fatty acids and cosolvent; IL-2-IL-2R interaction regions and interaction types derived from the X-ray structure; detailed interaction analysis between IL-2 and excipients from molecular dynamics simulations; and graphical representation of interaction profiles (PDF)

## References

[1] Lagasse H. A., Alexaki A., Simhadri V. L., Katagiri N. H., Jankowski W., Sauna Z. E., Kimchi-Sarfaty C. (2017). Recent advances in (therapeutic protein)
drug development. F1000Res..

[2] Mascarenhas-Melo F., Diaz M., Goncalves M. B. S., Vieira P., Bell V., Viana S., Nunes S., Paiva-Santos A. C., Veiga F. (2024). An Overview of Biosimilars-Development,
Quality, Regulatory Issues,
and Management in Healthcare. Pharmaceuticals.

[3] Yang Y., Lundqvist A. (2020). Immunomodulatory Effects of IL-2 and IL-15; Implications
for Cancer Immunotherapy. Cancers.

[4] Zhou Y., Quan G., Liu Y., Shi N., Wu Y., Zhang R., Gao X., Luo L. (2023). The application of
Interleukin-2 family cytokines in tumor immunotherapy research. Front. Immunol..

[5] Levin A. M., Bates D. L., Ring A. M., Krieg C., Lin J. T., Su L., Moraga I., Raeber M. E., Bowman G. R., Novick P. (2012). Exploiting
a natural conformational switch to engineer an interleukin-2
’superkine. Nature.

[6] Yuzhalin, A. E. ; Kutikhin, A. G. Interleukin-2 Superfamily and Cancer. In Interleukins in Cancer Biology; Academic Press, Yuzhalin, A. E. , Kutikhin, A. G. , Eds., 2015; pp 63–89.

[7] Anderson P. M., Sorenson M. A. (1994). Effects of route and formulation on clinical pharmacokinetics
of interleukin-2. Clin. Pharmacokinet..

[8] Oppenheim, J. J. ; Feldmann, M. ; Durum, S. K. Cytokine Reference: A Compendium of Cytokines and Other Mediators of Host Defense. Receptors; Academic Press, 2001.

[9] Klein C., Waldhauer I., Nicolini V., Dunn C., Freimoser-Grundschober A., Danny G., Boerman O., Nayak T., Herter S., Van Puijenbroek E. (2013). Novel Tumor-Targeted, Engineered IL-2
Variant (IL2v)-Based Immunocytokines For Immunotherapy Of Cancer. Blood.

[10] Charych D. H., Hoch U., Langowski J. L., Lee S. R., Addepalli M. K., Kirk P. B., Sheng D., Liu X., Sims P. W., VanderVeen L. A. (2016). NKTR-214, an Engineered
Cytokine with Biased
IL2 Receptor Binding, Increased Tumor Exposure, and Marked Efficacy
in Mouse Tumor Models. Clin. Cancer Res..

[11] Silva D. A., Yu S., Ulge U. Y., Spangler J. B., Jude K. M., Labao-Almeida C., Ali L. R., Quijano-Rubio A., Ruterbusch M., Leung I. (2019). De novo design of potent and selective mimics of IL-2
and IL-15. Nature.

[12] Bashir S., Fitaihi R., Abdelhakim H. E. (2023). Advances
in formulation and manufacturing
strategies for the delivery of therapeutic proteins and peptides in
orally disintegrating dosage forms. Eur. J.
Pharm. Sci..

[13] Moghaddasi F., Housaindokht M. R., Darroudi M., Bozorgmehr M. R., Sadeghi A. (2018). Soybean oil-based nanoemulsion
systems in absence and presence of curcumin: Molecular dynamics simulation
approach. J. Mol. Liq..

[14] Jensen C., Teng Y. (2020). Is It Time to Start
Transitioning From 2D to 3D Cell Culture?. Front.
Mol. Biosci..

[15] Saraiva D. P., Matias A. T., Braga S., Jacinto A., Cabral M. G. (2020). Establishment
of a 3D Co-culture With MDA-MB-231 Breast Cancer Cell Line and Patient-Derived
Immune Cells for Application in the Development of Immunotherapies. Front. Oncol..

[16] Arkin M. R., Randal M., DeLano W. L., Hyde J., Luong T. N., Oslob J. D., Raphael D. R., Taylor L., Wang J., McDowell R. S. (2003). Binding
of small molecules to an adaptive protein-protein
interface. Proc. Natl. Acad. Sci. U. S. A..

[17] Hyde J., Braisted A. C., Randal M., Arkin M. R. (2003). Discovery and characterization
of cooperative ligand binding in the adaptive region of interleukin-2. Biochemistry.

[18] Thanos C. D., Randal M., Wells J. A. (2003). Potent small-molecule binding to
a dynamic hot spot on IL-2. J. Am. Chem. Soc..

[19] Thanos C. D., DeLano W. L., Wells J. A. (2006). Hot-spot
mimicry of a cytokine receptor
by a small molecule. Proc. Natl. Acad. Sci.
U. S. A..

[20] Rickert M., Wang X., Boulanger M. J., Goriatcheva N., Garcia K. C. (2005). The structure of interleukin-2 complexed with its alpha
receptor. Science.

[21] Wang X., Rickert M., Garcia K. C. (2005). Structure of the
quaternary complex
of interleukin-2 with its alpha, beta, and gammac receptors. Science.

[22] Stauber D. J., Debler E. W., Horton P. A., Smith K. A., Wilson I. A. (2006). Crystal
structure of the IL-2 signaling complex: paradigm for a heterotrimeric
cytokine receptor. Proc. Natl. Acad. Sci. U.
S. A..

[23] Klein C., Waldhauer I., Nicolini V. G., Freimoser-Grundschober A., Nayak T., Vugts D. J., Dunn C., Bolijn M., Benz J., Stihle M. (2017). Cergutuzumab amunaleukin
(CEA-IL2v), a CEA-targeted IL-2 variant-based immunocytokine for combination
cancer immunotherapy: Overcoming limitations of aldesleukin and conventional
IL-2-based immunocytokines. OncoImmunology.

[24] Bamborough P., Hedgecock C. J., Richards W. G. (1994). The interleukin-2 and interleukin-4
receptors studied by molecular modelling. Structure.

[25] Mott H. R., Baines B. S., Hall R. M., Cooke R. M., Driscoll P. C., Weir M. P., Campbell I. D. (1995). The solution
structure of the F42A
mutant of human interleukin 2. J. Mol. Biol..

[26] McKay D. B. (1992). Response. Science.

[27] Jehle, S. ; Brenke, R. ; Vajda, S. ; Allen, K. N. ; Kozakov, D. Small molecular fragments bound to binding energy hot-spot in crystal contact interface of Interleukin-2; Worldwide Protein Data Bank, 2014.

[28] Arenas-Ramirez N., Zou C., Popp S., Zingg D., Brannetti B., Wirth E., Calzascia T., Kovarik J., Sommer L., Zenke G. (2016). Improved
cancer immunotherapy by a CD25-mimobody conferring
selectivity to human interleukin-2. Sci. Transl.
Med..

[29] Trotta E., Bessette P. H., Silveria S. L., Ely L. K., Jude K. M., Le D. T., Holst C. R., Coyle A., Potempa M., Lanier L. L. (2018). A human
anti-IL-2 antibody that potentiates
regulatory T cells by a structure-based mechanism. Nat. Med..

[30] Wang Y., Wang G., Li Y., Zhu Q., Shen H., Gao N., Xiao J. (2020). Structural insights
into secretory immunoglobulin A
and its interaction with a pneumococcal adhesin. Cell Res..

[31] Lopes J. E., Fisher J. L., Flick H. L., Wang C., Sun L., Ernstoff M. S., Alvarez J. C., Losey H. C. (2020). ALKS 4230: a novel
engineered IL-2 fusion protein with an improved cellular selectivity
profile for cancer immunotherapy. J. Immunother
Cancer.

[32] Karakus U., Sahin D., Mittl P. R. E., Mooij P., Koopman G., Boyman O. (2020). Receptor-gated IL-2
delivery by an anti-human IL-2
antibody activates regulatory T cells in three different species. Sci. Transl. Med..

[33] Kim J., Lee J. Y., Park S. Y., Lee Y. J., Kim M. S. (2021). Crystal
structure of human interleukin-2 in complex with TCB2, a new antibody-drug
candidate with antitumor activity. OncoImmunology.

[34] Ptacin J. L., Caffaro C. E., Ma L., San Jose Gall K. M., Aerni H. R., Acuff N. V., Herman R. W., Pavlova Y., Pena M. J., Chen D. B. (2021). An
engineered IL-2 reprogrammed
for anti-tumor therapy using a semi-synthetic organism. Nat. Commun..

[35] Ren J., Chu A. E., Jude K. M., Picton L. K., Kare A. J., Su L., Montano
Romero A., Huang P. S., Garcia K. C. (2022). Interleukin-2
superkines by computational design. Proc. Natl.
Acad. Sci. U. S. A..

[36] Goryacheva E. A., Artemyev I. V., Pletneva N. V., Pletnev V. Z. (2023). Three-Dimensional
Structure of Fab Fragment of Monoclonal Antibody LNKB-2 Complexed
with Antigenic Nonaptide from Human Interleukin-2. Bioorg. Chem..

[37] Jumper J., Evans R., Pritzel A., Green T., Figurnov M., Ronneberger O., Tunyasuvunakool K., Bates R., Zidek A., Potapenko A. (2021). Highly accurate protein structure prediction with AlphaFold. Nature.

[38] Schrödinger Release 2021–2: Maestro Schrödinger; LLC: New York, N. Y., 2021.

[39] Schrödinger Release 2021–2: Schrödinger Suite 2021–2 Protein Preparation Wizard; Epik, S., LLC, New York, NY, 2021; Impact, Schrödinger, LLC, New York, NY, 2021; Prime, Schrödinger, LLC, New York, NY, 2021.

[40] Schrödinger Release 2021–2: LigPrep Schrödinger; LLC: New York, N. Y., 2021.

[41] Schrödinger Release 2021–2: Desmond Molecular Dynamics System D. E. Shaw Research New York, N. Y.; Maestro-Desmond Interoperability Tools, Schrödinger, New York, N.Y., 2021.

[42] Ding S., Anton N., Akram S., Er-Rafik M., Anton H., Klymchenko A., Yu W., Vandamme T. F., Serra C. A. (2017). A new method
for the formulation of double nanoemulsions. Soft Matter.

[43] Ali F. R., Shoaib M. H., Ali S. A., Yousuf R. I., Siddiqui F., Raja R., Jamal H. S., Saleem M. T., Ahmed K., Imtiaz M. S. (2022). A nanoemulsion
based transdermal delivery of
insulin: Formulation development, optimization, in-vitro permeation
across Strat-M® membrane and its pharmacokinetic/pharmacodynamic
evaluation. J. Drug Delivery Sci. Technol..

[44] Żarska M., Dzida M., Apelblat A. (2017). Surface tensions and
densities of
concentrated aqueous solutions of citric acid. J. Mol. Liq..

[45] Timur B., Yilmaz Usta D., Teksin Z. S. (2022). Investigation of the effect of colloidal
structures formed during lipolysis of lipid-based formulation on exemestane
permeability using the in vitro lipolysis-permeation model. J. Drug Delivery Sci. Technol..

[46] Dehghan R., Beig Parikhani A., Zeinali S., Shokrgozar M., Amanzadeh A., Ajdary S., Ahangari Cohan R., Talebkhan Y., Behdani M. (2022). Efficacy and antitumor activity of
a mutant type of interleukin 2. Sci. Rep.

[47] Belfiore L., Aghaei B., Law A. M. K., Dobrowolski J. C., Raftery L. J., Tjandra A. D., Yee C., Piloni A., Volkerling A., Ferris C. J. (2021). Generation
and analysis
of 3D cell culture models for drug discovery. Eur. J. Pharm. Sci..

[48] Rausch M., Blanc L., De Souza
Silva O., Dormond O., Griffioen A. W., Nowak-Sliwinska P. (2021). Characterization of Renal Cell Carcinoma Heterotypic
3D Co-Cultures with Immune Cell Subsets. Cancers.

[49] Haddadzadegan S., Dorkoosh F., Bernkop-Schnurch A. (2022). Oral delivery
of therapeutic peptides
and proteins: Technology landscape of lipid-based nanocarriers. Adv. Drug Deliv Rev..

