# Supplementary material for: Screening Oil Components for Interleukin-2-Loaded Lipid-Based Formulations with Molecular Dynamics, In Vitro Characterization, and Cell Culture Evaluation
Source: ACS Omega. 2026 Feb 26;11(9):15028–43. doi: 10.1021/acsomega.5c12065 (PMC12980213; doi:10.1021/acsomega.5c12065)
Supplement: Supplementary file 1 [file ao5c12065_si_001.pdf]

Screening oil components for interleukin-2 loaded lipid-based formulations with molecular dynamics, *in vitro* characterization, and cell culture evaluation

*Seval Olgac[a], Abdurrahman Olgac[b,c], Gamze Varan[d], Zeynep Safak Teksin[a]**

[a]Department of Pharmaceutical Technology, Faculty of Pharmacy, Gazi University, 06560 Ankara, Türkiye

[b]Department of Pharmaceutical Chemistry, Faculty of Pharmacy, Gazi University, 06560 Ankara, Türkiye

[c]Laboratory of Molecular Modeling, Evias Pharmaceutical R&D Ltd, Gazi Teknopark, 06830 Ankara, Türkiye

[d]Department of Vaccine Technology, Vaccine Institute, Hacettepe University, 06100 Ankara, Türkiye.

* E-mail: zsteksin@gazi.edu.tr

Table S1. Excipients' characteristics according to technical data sheets

	Characteristic	Specification
**Labrafac Lipophile WL 1349**	Caproic acid (C6)	≤2.0%
	Caprylic acid (C8)	50.0 to 80.0 %
	Capric acid (C10)	20.0 to 50.0 %
	Lauric acid (C12)	≤3.0%
	Myristic acid (C14)	≤1.0%
**Labrasol ALF**	Caproic acid (C6)	≤2.0%
	Caprylic acid (C8)	50.0 to 80.0 %
	Capric acid (C10)	20.0 to 50.0 %
	Lauric acid (C12)	≤3.0%
	Myristic acid (C14)	≤1.0%
**Lauroglycol 90**	Caprylic acid (C8)	≤0.5%
	Capric acid (C10)	≤2.0%
	Lauric acid (C12)	≥95.0 %
	Myristic acid (C14)	≤3.0%
	Palmitic acid (C16)	≤1.0%
**Transcutol HP**	Diethylene glycol monoethyl ether	≥99.900 %
**Maisine CC**	Palmitic acid (C16)	4.0 to 20.0 %
	Stearic acid (C18)	≤6.0%
	Oleic acid (C18:1)	10.0 to 35.0 %
	Linoleic acid (C18:2)	≥50.0%
	Linoleic acid (C18:3)	≤2.0%
	Arachidic acid (C20)	≤1.0%
	Eicosenoic acid (C20:1)	≤1.0%
**Peceol**	Palmitic acid (C16)	≤12.0%
	Stearic acid (C18)	≤6.0%
	Oleic acid (C18:1)	≥60.0%
	Linoleic acid (C18:2)	≤35.0%
	Linoleic acid (C18:3)	≤2.0%
	Arachidic acid (C20)	≤2.0%
	Eicosenoic acid (C20:1)	≤2.0%
**Capryol 90**	Caprylic acid (C8)	>= 90.0 %
	Capric acid (C10)	<= 3.0 %
	Lauric acid (C12)	<= 3.0 %
	Myristic acid (C14)	<= 3.0 %
	Palmitic acid (C16)	<= 1.0 %

**10% and above have been taken into consideration while building simulation systems.*

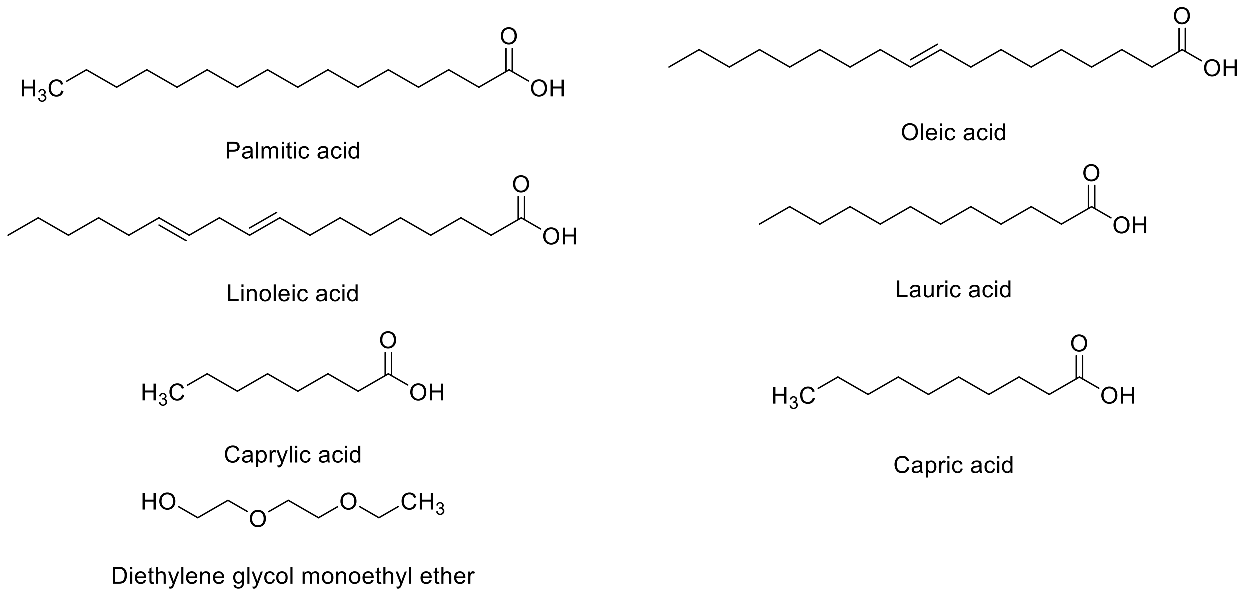

Figure S1. The molecular structures of palmitic acid, oleic acid, linoleic acid, lauric acid, caprylic acid, capric acid, and diethylene glycol monoethyl ether.

Table S2. Interaction regions and interaction types of IL-2 with the subunits of IL-2R, obtained from the X-ray structure (PDB id: 2ERJ)

**IL-2**	**IL-2R**	**Observed Interaction Type**
Lys 35	α	Nonpolar Interaction
Thr 37	α	H-Bonds
Arg 38	α	H-Bonds, Salt Bridge
Thr 41	α	H-Bonds
Phe 42	α	Nonpolar Interaction
Lys 43	α	H-Bonds
Phe 44	α	Nonpolar Interaction
Tyr 45	α	Nonpolar Interaction, H-Bonds, π-Cation Interaction
Glu 61	α	H-Bonds, Salt Bridge
Glu 62	α	Salt Bridge
Lys 64	α	H-Bonds

Pro 65	α	Nonpolar Interaction
Glu 68	α	H-Bonds
Leu 72	α	Nonpolar Interaction
Cys 105	α	H-Bonds
Leu 12	β	Nonpolar Interaction
Gln 13	β	Nonpolar Interaction
Glu 15	β	Salt Bridge
His 16	β	Nonpolar Interaction
Asp 20	β	H-Bonds, Salt Bridge
Asp 84	β	Salt Bridge
Asn 88	β	H-Bonds
Ile 92	β	Nonpolar Interaction
Glu 95	β	Nonpolar Interaction, Salt Bridge
Gln 22	γ	H-Bonds
Thr 123	γ	Nonpolar Interaction
Gln 126	γ	Nonpolar Interaction, H-Bonds
Ser 130	γ	H-Bonds

Table S3. Interaction analysis of excipient and IL-2

**Oleic acid**	**Oleic acid 1**	**Lys 49** (19%, Hydrophilic); **Glu 116** (12%, Hydrophilic); **Asn 119** (12.5%, Hydrophilic); **Arg 120** (15%, Hydrophilic)	**Capryol 90**	**Caprylic acid 1**	**Tyr 31** (25%, Hydrophobic, Hydrophilic); **Lys 35** (80%, Hydrophilic); **Arg 38** (80%, Hydrophilic); **Ser 75** (25%, Hydrophilic)
	**Oleic acid 2**	**Lys 8** (12%, Hydrophilic)		**Caprylic acid 2**	**Lys 32**(10%, Hydrophilic); **Lys 49** (35%, Hydrophilic); **Arg 120** (30%, Hydrophilic)
	**Oleic acid 3**	**Asn 33** (30%, Hydrophilic); **Thr 37** (20%, Hydrophilic); **Ile 114** (25%, Hydrophobic); **Val 115** (20%, Hydrophobic, Hydrophilic)		**Caprylic acid 3**	**Asn 30** (15%, Hydrophilic); **Lys 76** (>75%, Hydrophilic); **Phe 78** (30%, Hydrophobic, Hydrophilic); **His 79** (95%, Hydrophilic)
	**Oleic acid 4**	**Lys 49** (10%, Hydrophilic)		**Caprylic acid 4**	**Arg 38** (60%, Hydrophilic)

Labrafac Lipophile WL 1349	Caprylic acid 1	**Arg 120** (12%, Hydrophilic)	Lauroglycol 90	Lauric acid 1	**Asn 30** (10%, Hydrophilic); **Lys 35** (20%, Hydrophilic); **Arg 38** (40%, Hydrophilic); **Asn 77** (15%, Hydrophilic)
	Caprylic acid 2	**Lys 35** (95%, Hydrophilic); **Arg 38** (70%, Hydrophilic), **Lys 76** (40%, Hydrophilic)		Lauric acid 2	**Lys 35** (10%, Hydrophilic); **Arg 38** (100%, Hydrophilic)
	Capric acid 1	**Ala 50** (10%, Hydrophilic); **Thr 51** (30%, Hydrophilic); **Glu 52** (25%, Hydrophilic)		Lauric acid 3	<10%
	Capric acid 2	**Arg 38** (95%, Hydrophilic)		Lauric acid 4	**Lys 8** (10%, Hydrophilic); **Lys 9** (10%, Hydrophilic); **Pro 47** (10%, Hydrophilic); **Ala 50** (10% Hydrophilic,); **Arg 120** (80%, Hydrophilic)
Maisine	Oleic acid	**Thr 7** (13%, Hydrophilic); **Lys 8** (15%, Hydrophilic); **Lys 54** (14%, Hydrophilic)	Labrasol	Capric acid	**Lys 48** (10%, Hydrophilic); **Arg 120** (40%, Hydrophilic)
	Palmitic acid	**Asn 33** (30%, Hydrophilic); **Thr 37** (10%, Hydrophilic); **Leu 40** (10%, Hydrophobic, Hydrophilic); **Ile 114** (10%, Hydrophobic)		Caprylic acid 1	**Lys 32** (10%, Hydrophilic); **Lys 35** (12%, Hydrophilic); **Arg 38** (10%, Hydrophilic); **Lys 76** (10%, Hydrophilic)
	Linoleic acid 1	**Arg 38** (17.5%, Hydrophilic); **Leu 53** (15%, Hydrophobic, Hydrophilic); **Lys 54** (12.5%, Hydrophilic)		Caprylic acid 2	**Tyr 31** (10%, Hydrophobic, Hydrophilic); **Lys 35** (60%, Hydrophilic); **Arg 38** (80%, Hydrophilic); **Leu 72** (15%, Hydrophilic, Hydrophobic); **Lys 76** (30%, Hydrophilic)
	Linoleic acid 2	**Lys 9** (13%, Hydrophilic); **Pro 47** (13%, Hydrophobic); **Arg 120** (14%, Hydrophilic)		Caprylic acid 3	**Arg 38** (10%, Hydrophilic); **Arg 120** (20%, Hydrophilic)
Peceol	Palmitic acid	**Lys 49** (14%, Hydrophilic); **Arg 120** (10%, Hydrophilic)	Transcutol HP	Diethylene glycol monoethyl ether1	<10%
	Linoleic acid	**Arg 38** (20%, Hydrophilic)		Diethylene glycol monoethyl ether2	<10%
	Oleic acid 1	**Lys 35** (80%, Hydrophilic); **Arg 38** (80%, Hydrophilic); **Phe 42** (20%, Hydrophobic); **Leu 72** (30%, Hydrophilic, Hydrophobic); **Gln 74** (20%, Hydrophilic)		Diethylene glycol monoethyl ether3	**Pro 47** (25%, Hydrophilic, Hydrophobic); **Arg 120** (50%, Hydrophilic); **Trp 121** (10%, Hydrophobic, Hydrophilic)
	Oleic acid 2	**Lys 49** (25%, Hydrophilic); **Ala 50** (25%, Hydrophilic); **Thr 51**(15%, Hydrophilic); **Arg 120** (40%, Hydrophilic); **Trp 121** (10%, Hydrophobic)		Diethylene glycol monoethyl ether4	<10%

**Interactions of 10% and above between excipients and IL-2 have been reported.*

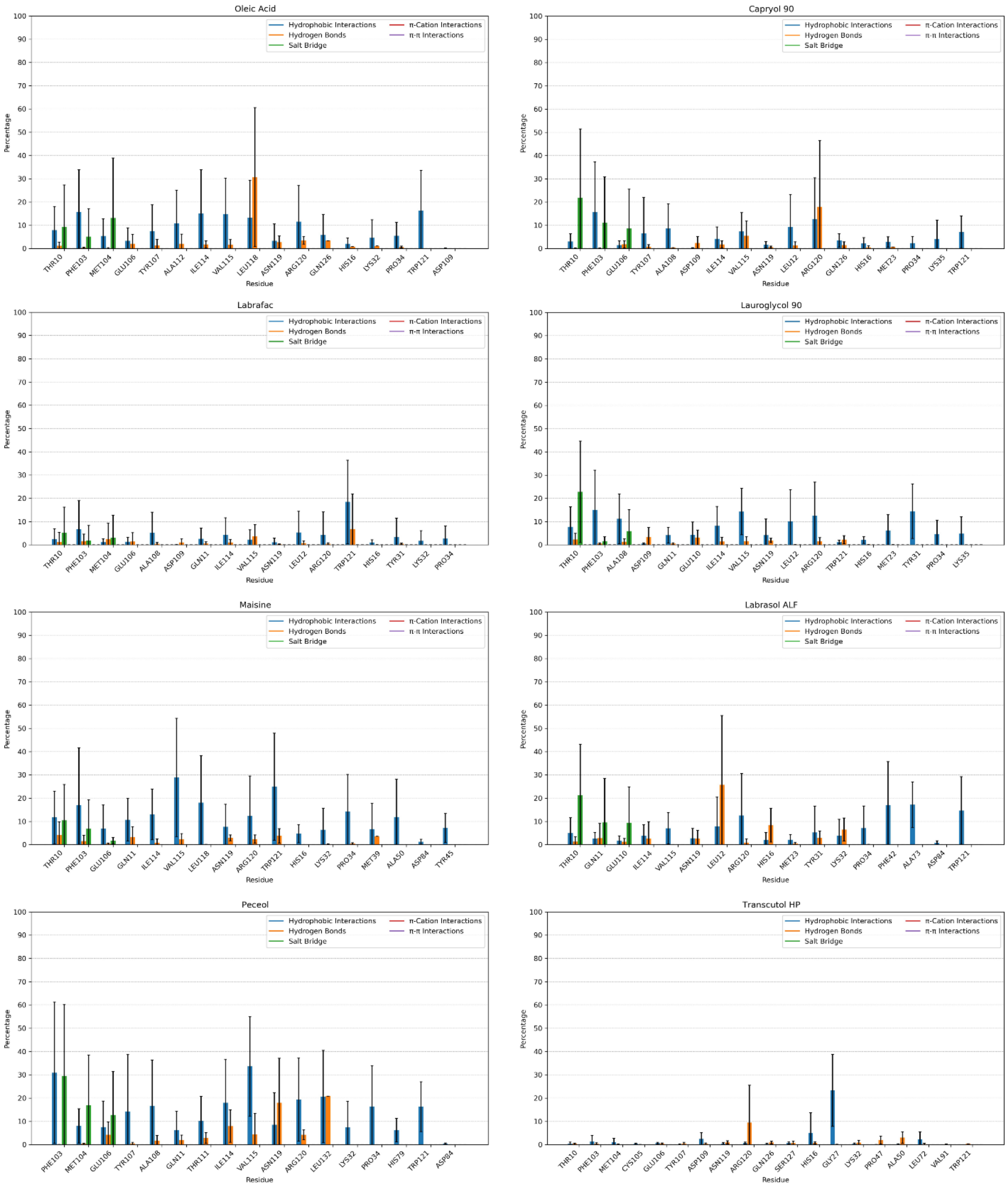

Figure S2. Cell-bar plot representation of the interaction analysis between IL-2 and excipients.
